# Transcriptome analysis reveals ginsenosides biosynthetic genes, microRNAs and simple sequence repeats in *Panax ginseng* C. A. Meyer

**DOI:** 10.1186/1471-2164-14-245

**Published:** 2013-04-11

**Authors:** Chunfang Li, Yingjie Zhu, Xu Guo, Chao Sun, Hongmei Luo, Jingyuan Song, Ying Li, Lizhi Wang, Jun Qian, Shilin Chen

**Affiliations:** 1 Chinese Academy of Medical Sciences & Peking Union Medical College, Institute of Medicinal Plant Development, Beijing, 100094, China; 2 China Academy of Chinese Medical Sciences, Institute of Chinese Materia Medica, Beijing, 100700, China

**Keywords:** Expressed sequence tag, microRNA, Simple sequence repeats, Ginsenoside, *Panax ginseng* C. A. Meyer

## Abstract

**Background:**

*Panax ginseng* C. A. Meyer is one of the most widely used medicinal plants. Complete genome information for this species remains unavailable due to its large genome size. At present, analysis of expressed sequence tags is still the most powerful tool for large-scale gene discovery. The global expressed sequence tags from *P. ginseng* tissues, especially those isolated from stems, leaves and flowers, are still limited, hindering in-depth study of *P. ginseng*.

**Results:**

Two 454 pyrosequencing runs generated a total of 2,423,076 reads from *P. ginseng* roots, stems, leaves and flowers. The high-quality reads from each of the tissues were independently assembled into separate and shared contigs. In the separately assembled database, 45,849, 6,172, 4,041 and 3,273 unigenes were only found in the roots, stems, leaves and flowers database, respectively. In the jointly assembled database, 178,145 unigenes were observed, including 86,609 contigs and 91,536 singletons. Among the 178,145 unigenes, 105,522 were identified for the first time, of which 65.6% were identified in the stem, leaf or flower cDNA libraries of *P. ginseng*. After annotation, we discovered 223 unigenes involved in ginsenoside backbone biosynthesis. Additionally, a total of 326 potential cytochrome P450 and 129 potential UDP-glycosyltransferase sequences were predicted based on the annotation results, some of which may encode enzymes responsible for ginsenoside backbone modification. A BLAST search of the obtained high-quality reads identified 14 potential microRNAs in *P. ginseng*, which were estimated to target 100 protein-coding genes, including transcription factors, transporters and DNA binding proteins, among others. In addition, a total of 13,044 simple sequence repeats were identified from the 178,145 unigenes.

**Conclusions:**

This study provides global expressed sequence tags for *P. ginseng*, which will contribute significantly to further genome-wide research and analyses in this species. The novel unigenes identified here enlarge the available *P. ginseng* gene pool and will facilitate gene discovery. In addition, the identification of microRNAs and the prediction of targets from this study will provide information on gene transcriptional regulation in *P. ginseng*. Finally, the analysis of simple sequence repeats will provide genetic makers for molecular breeding and genetic applications in this species.

## Background

*Panax ginseng* C. A. Meyer is a widely used medicinal plant with multiple clinical and pharmacological effects related to cancer, diabetes and cardiovascular disease. It also promotes immune and central nervous system function as well as relieving stress [1]. The major bioactive components of *P. ginseng* are the ginsenosides, a group of dammarane- and oleanane-type triterpenoid saponins. The total ginsenoside content is highest in the flower, followed by the root, leaf and stem [2]. The large size and high complexity of the *P. ginseng* genome, which is reportedly tetraploid and ~3.2 Gb in size [3], has made it difficult to obtain a complete genomic sequence for this species. Many researchers have obtained genomic information for *P. ginseng* by employing expressed sequence tags (ESTs), which are considered an efficient tool for gene discovery, especially in plants lacking an assembled genome [4]. Previous studies have generated ESTs derived from *P. ginseng* roots, rhizomes, seeds and leaves using the Sanger method [5-8]. However, as this method has a high cost and is very time consuming, only 17,773 ESTs obtained using this technique have been deposited in NCBI to date. Next-generation sequencing (NGS) technologies provide a rapid and economical way to sequence extremely large amounts of genetic material [9,10]. For example, Chen et al. generated 217,529 ESTs from 11-year-old *P. ginseng* roots via 454 sequencing [11]. Given that different tissues exhibit specific gene expression patterns, it is necessary to obtain the global transcriptome of other tissues to obtain full genomic information for *P. ginseng*.

ESTs are considered to represent a reliable source of data for predicting microRNAs (miRNAs) and their targets, especially in species without complete genome information [12]. miRNAs are important regulators in a wide range of developmental processes in plants, including cell proliferation, the stress response, metabolism, inflammation and signal transduction [13,14]. miRNAs have been identified successfully from plant EST databases based on sequence conservation and characteristic miRNA features [15-17]. However, miRNAs have not yet been identified from *P. ginseng*.

Simple sequence repeats (SSRs), also termed microsatellites, are nucleotide motifs consisting of tandem repeats of two to six base pairs. It is more likely for SSRs from ESTs (EST-SSRs) to be tightly linked to specific gene functions and perhaps even play a direct role in controlling important agronomic traits [18]. To date, only 251 SSRs have been identified in *P. ginseng*[19].

In the present study, we globally sequenced the transcriptomes of the roots, stems, leaves and flowers of 4-year-old *P. ginseng* plants. Novel and tissue-specific *P. ginseng* unigenes were obtained. Furthermore, our database includes all of the genes encoding enzymes involved in ginsenoside backbone biosynthesis and modification. Based on the obtained unigenes, we also identified 14 potential *P. ginseng* miRNAs and 100 of their potential target genes. Moreover, a total of 13,044 EST-SSRs were identified from the *P. ginseng* unigene dataset, which will facilitate marker-assisted breeding of *P. ginseng*.

## Results and discussion

### Sequencing and *de novo* assembly

To characterize the transcriptome of *P. ginseng* and generate expression profiles, we sequenced cDNA samples from four *P. ginseng* tissues (root, stem, leaf and flower) using a Roche/454 GS-FLX (Titanium) pyrosequencing machine. One half run was performed for each sample, yielding approximately 2.42 million raw reads, ultimately totaling ~1.01 billion base pairs (bp). Of these ESTs, 70.2% were over 400 bp in length. The size distributions of the raw reads from the four samples are shown in Additional file 1. To acquire high-quality reads, we filtered out adapter sequences and reads that were shorter than 50 bp. The high-quality reads from each sample were then used to build a *de novo* assembly using GS De NovoAssembler software, v2.6, both for each tissue individually and for all tissues as a group. The size distributions of the contigs from each tissue are presented in Additional file 2. After assembling the reads from all four tissues together, the generated contigs ranged from 100 to 7,858 bp, with an average size of 468.7 bp, while the size of the singletons ranged from 100 to 691 bp, averaging 382.9 bp. We obtained 178,145 unique sequences, totaling approximately 75.6 Mb. All of the high-quality reads generated in this study have been deposited at NCBI and can be accessed in the Sequence Read Achieve (SRA) Sequence Database under project accession number SRP015263. This Transcriptome Shotgun Assembly project has been deposited at DDBJ/EMBL/GenBank under accession GAAG00000000. The version described in this paper is the first version, GAAG01000000. A summary of the 454 sequencing and assembly results for the four tissues is shown in Additional file 3, and the data for together assembly is presented in Table 1.

**Table 1:** **Summary of the total 454 sequencing and the assembly results for *P. ginseng *four tissues**

	**No. of sequences**	**No. of bases**
High-quality reads	2,423,076	980,165,285
Average high-quality read length (bp)	404.5	
Reads used in assembly	2,151,832	863,992,539
Number of contigs ≥100 bp	86,609	40,589,347
Average length of contigs (bp)	468.7	
Range of contig lengths (bp)	100-7,858	
Number of singletons ≥100 bp	91,536	35,032,649
Average length of singletons (bp)	382.9	
Range of singleton lengths (bp)	100-691	
Number of unigenes (contigs and singletons)	178,145	
Total coverage (bp)	75,621,996	

The current *P. ginseng* EST library found in the TSA database contains 15,357 ESTs from 11-year-old root tissue [11]. These ESTs are included in the 33,903 homologous unigenes revealed in the 4-year-old *P. ginseng* root transcriptome examined in the present study. Furthermore, 82,666 novel *P. ginseng* root unigenes were discovered in this study, some of which may be specifically expressed in 4-year-old roots. Whereas there are 32,441 ESTs in the NCBI database that were obtained via 454 and Sanger sequencing, 72,623 homologous genes and 105,522 novel unigenes were discovered in the present study. The large quantity of novel unique sequences identified in this study constitutes a powerful resource for *P. ginseng* researchers.

### Functional annotation and candidate genes encoding enzymes involved in the biosynthesis of ginsenosides

To obtain the most informative and complete annotation, all of the contigs from roots, stems, leaves and flowers were annotated separately. The numbers and percentages of the annotated unique sequences are presented in Additional file 4. In total, 94,535 unique sequences presented at least one significant match in the public databases. The remaining unigenes that were not annotated appeared to be either *P. ginseng*-specific genes or homologous genes with unknown functions in other species.

**Figure 1:**
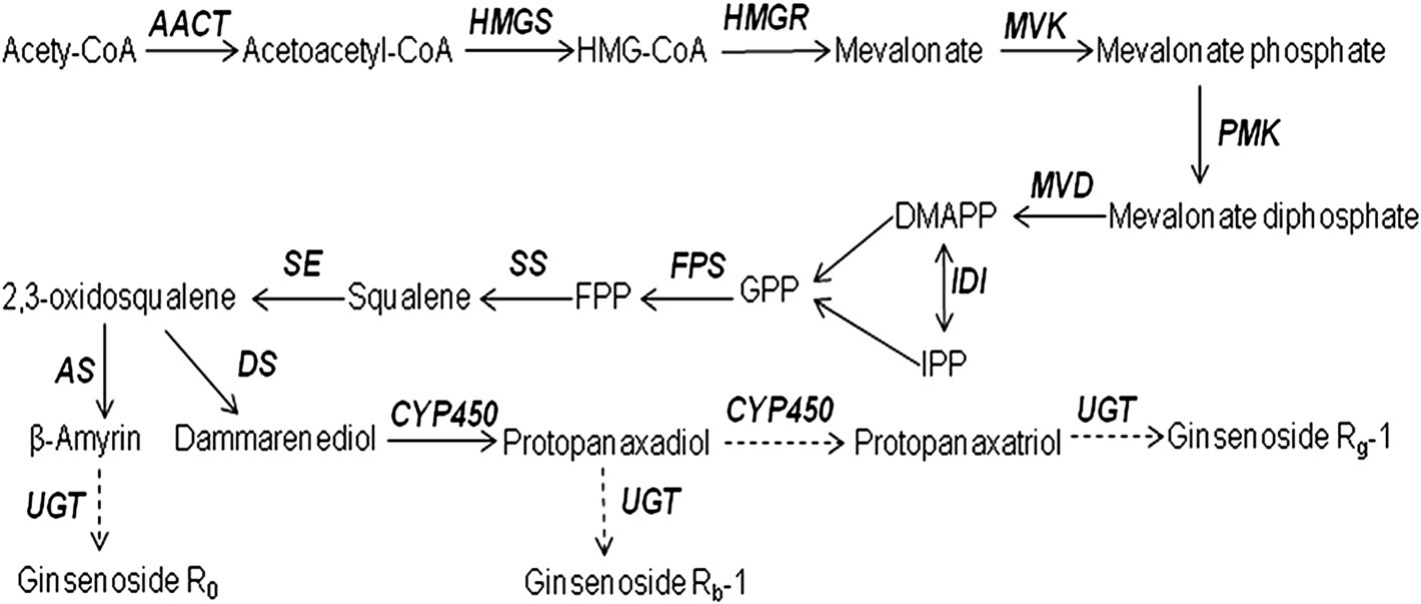
**Putative ginsenoside biosynthesis pathway.** Candidate genes identified in this study are shown in bold. Acetyl-CoA acetyltransferase (*AACT*), HMG-CoA synthase (*HMGS*), HMG-CoA reductase (*HMGR*), mevalonate kinase (*MVK*), phosphomevalonate kinase (*PMK*), mevalonate diphosphate decarboxylase (*MVD*), isopentenyl diphosphate isomerase (*IDI*), geranylgeranyl pyrophosphate synthase (*GPS*), farnesyl diphosphate synthase (*FPS*), squalene synthase (*SS*), squalene epoxidase (*SE*), amyrin synthase (*AS*), dammarendiol synthase (*DS*), *UDP* glycosyltransferase (*UGT*) and cytochrome P450 (*CYP450*).

Based on the annotation results, transcripts encoding all of the known enzymes involved in ginsenoside biosynthesis and modification were identified in our dataset (Figure 1). In most cases, multiple unigenes were annotated as corresponding to the same enzyme (Additional file 5). Such unigenes may represent different fragments of a single transcript or different members of a gene family.

Chen et al. [11] analyzed the transcriptome of 11-year-old *P. ginseng* roots and discovered many genes involved in ginsenoside biosynthesis. However, several genes encoding key enzymes involved in ginsenoside skeleton biosynthesis were absent, such as *mevalonate kinase* (*MVK*), *geranylgeranyl pyrophosphate synthase* (*GPS*), *amyrin synthase* (*AS*) and *dammarendiol synthase* (*DS*). From the global tissue transcriptome analysis, we obtained higher coverage genetic information and more candidate genes for further analysis. *MVK*, *GPS* and *DS* were found in our 4-year-old root cDNA library (Table 2). This difference of results may be due to these three enzymes being actively expressed in the 4-year-old *P. ginseng* root, but expressed only at a low level in the 11-year-old *P. ginseng* root, or to the high coverage of the 4-year-old root transcriptome. *AS* was absent in both the 4- and 11-year-old *P. ginseng* roots but was found in the 4-year-old *P. ginseng* stems, leaves and flowers in the transcriptome database. This result may indicate that the biosynthsis of oleanane-type ginsenosides might be actived in the stem, leaf and flower of 4-year-old *P. ginseng*, but not in the root of the 4- and 11-year-old *P. ginseng*.

**Table 2:** **Comparison of the unigene numbers from tissues of 4- and 11-year-old *P. ginseng***

**Gene name**	**EC number**	**Unigene number**		
		**Four tissues of 4-year old plant**	**4-year-old root**	**11-year-old root**
*AACT*	2.3.1.9	18	5	4
*HMGS*	2.3.1.10	8	1	2
*HMGR*	1.1.1.34	32	5	2
*MVK*	2.7.1.36	9	3	0
*PMK*	2.7.4.2	16	8	2
*MVD*	4.1.1.33	11	3	1
*IDI*	5.3.3.2	15	1	2
*GPS*	2.5.1.29	45	2	0
*FPS*	2.5.1.1 2.5.1.10	14	2	3
*SS*	2.5.1.21	22	1	1
*SE*	1.14.99.7	21	3	1
*AS*		6	0	0
*DS*		16	1	0

Specific CYP450s catalyze the conversion of dammarenediol-II or β-amyrin to various ginsenosides. Han et al. identified the involvement of CYP716A47 in the hydroxylation of the C-12 position to yield protopanaxadiol [20]. In this study, 326 putative CYP450 unigenes were identified, including the *CYP716A47* gene. Based on our CYP450 pool, further research will be performed to identify other CYP450s that may participate in ginsenoside biosynthesis in *P. ginseng*. Glycosylation, the transfer of activated saccharides to an aglycone substrate, is the predominant type of modification that occurs in the last step of ginsenoside biosynthesis. Glycosylation of dammarane- and oleanane-type aglycones is required for ginsenoside bioactivity. In this study, 129 putative UDP glycosyltransferase (UGT) unigenes were found in the *P. ginseng* transcriptome, of which 6 showed a high sequence similarity to the candidate glucosidase genes identified in *P. quinquefolius* by Sun et al. [21]. These putative *P. ginseng UGT*s included contig89150, contig89599, contig48582, contig18298, contig76094 and contig72547 from the database derived from assembling all tissues simultaneously. The roles of these candidate *UGT* unigenes in ginsenoside biosynthesis need to be further characterized.

### Comparative and Gene Ontology analyses of *P. ginseng* root, stem, leaf and flower unigenes

**Figure 2:**
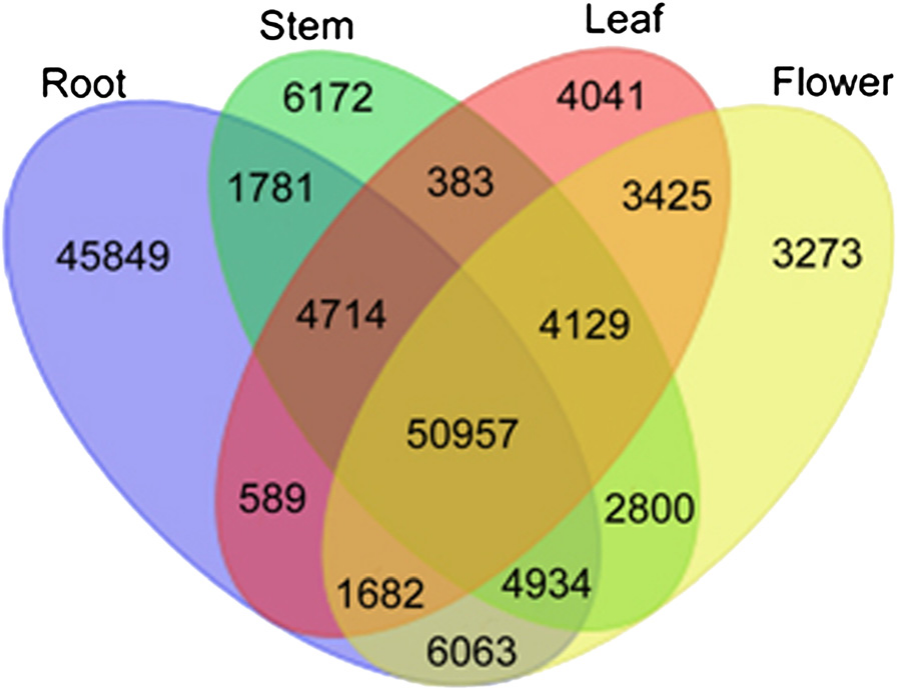
**Venn diagram of the unigenes in the roots, stems, leaves and flowers of *P. ginseng*.** Venn diagram showing the overlapping unigenes from *P. ginseng*: root, stem leaf and flower. A total of 50,957 unigenes were found in all tissues, whereas some unigenes could only be found in distinct tissue (root 45,849, stem 6,172, leaf 4,041 and flower 3,273), and others overlapped in two or even three tissues.

Unigene sequences of the *P. ginseng* four tissues were compared with each other and was shown by a Venn diagram (Figure 2). The four tissues shared 50,957 unigenes, which likely include housekeeping genes playing key roles in *P. ginseng*. The number of unigenes only can be found in the database of each tissue was 45,849 for the root, 6,172 for the stem, 4,041 for the leaf and 3,273 for the flower. The number of unigenes which can only be found in root database was highest among the four tissues, maybe because that the root expressed more genes than the other three tissues. The unigenes only found in the stem, leaf and flower databases corresponded to 65.6% of the novel genes discovered in our study and might represent genes controlling development in different tissues.

**Figure 3:**
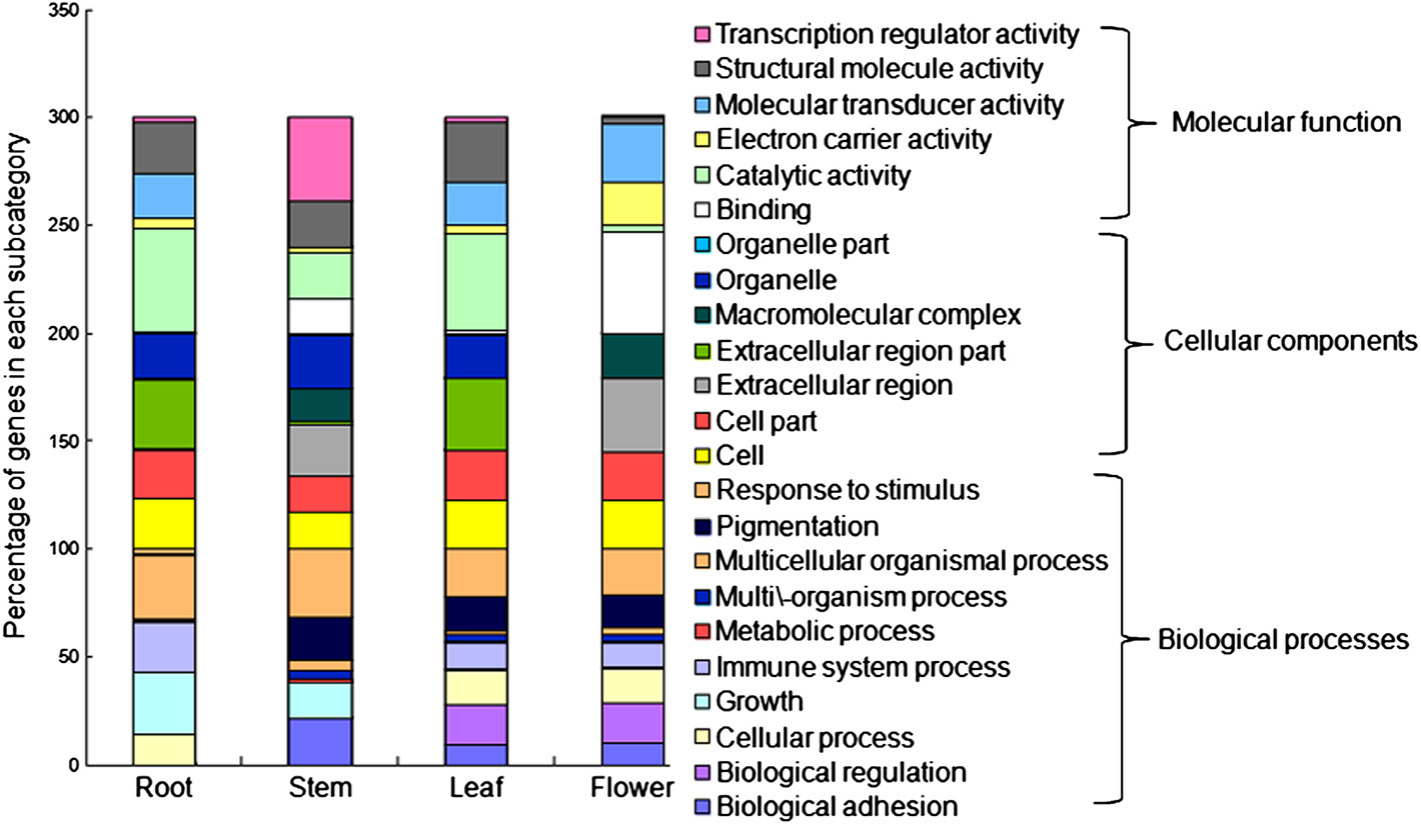
**Functional classification of unigenes in the four *P. ginseng *tissues based on Gene Ontology categories.** Unique sequences were classified into 23 gene ontology categories under three major categories: cellular components, molecular functions and biological processes.

Gene Ontology (GO) is widely used to standardize the representation of genes across species and to provide a controlled vocabulary of terms for describing gene products [22]. The contigs from four tissues were assigned GO terms based on BlastP searches against sequences with products whose functions were previously identified. These GO terms were summarized into three main GO categories (biological process, cellular component and molecular function) according to the standard GO terms and 23 sub-categories (Figure 3). In each tissue, the biological process category comprised the majority of GO annotations, followed by the cellular component and molecular function categories. The percentage of each sub-category in each tissue was quite different. Notably, within the biological process category in the root transcriptome, multicellular organismal process and growth were the most dominant subcategories, reflecting the rapid growth occurring in the root. In the other three tissues, the dominant subcategory was response to stimulus. For the cellular component category, the most highly represented subcategory in roots and leaves was extracellular region part, while in stems, it was organelle and in flowers, it was extracellular region. Under the molecular function category, the main subcategory in roots and leaves was catalytic activity, while it was transcription regulator activity in stems and binding in flowers. These GO annotations represent a general gene expression profile signature of the different tissues of *P. ginseng* and demonstrate that the genes expressed in these different tissues encode diverse structural, regulatory and stress response proteins.

### Analysis of the predominant transcripts in *P. ginseng* roots, stems, leaves and flowers

The abundance of particular transcripts within a specific tissue provides clues about the biological processes occurring there. The most highly expressed genes observed in each cDNA library are listed in Table 3. The genes encoding catalase and superoxide dismutase were present in all four cDNA libraries and presented particularly high levels in the root. Catalase and superoxide dismutase are two antioxidant enzymes that play key roles in antioxidant defense systems and in the protection of plant cells against oxidative damage caused by reactive oxygen species [23]. Other abundant transcripts in the root-derived library encoded a latex-like protein, ribonuclease-like storage protein, 1,4-alpha-glucan branching enzyme and some proteins with unknown functions, which could be *P. ginseng*-specific proteins.

**Table 3:** **Predominant transcripts in the cDNA libraries generated from *P. ginseng *roots, stems, leaves and flowers**

**Unigene ID**	**Subject**	**Annotation**	**No. of reads**
**Root**			
contig03234	vvi:100267971	Hypothetical protein	946
contig03264	vvi:100267971	Hypothetical protein	921
contig08673	sp|O24339|	Catalase	904
contig 08301	sp|P24669	Superoxide dismutase	872
contig01891	gb|ACH72969.1|	Latex-like protein	862
contig02117	sp|P83618|	Ribonuclease-like storage protein	846
contig08057	ref|XP_002467304.1|	Hypothetical protein	838
contig08066	ref|XP_002488947.1|	Hypothetical protein	788
contig08059	pop:POPTR_265927	Hypothetical protein	780
contig09140	vvi:100257371	1,4-alpha-glucan branching enzyme	776
**Stem**			
contig01514	sp|Q9C8U9|	Phloem protein 2-like protein	993
contig01413	gb|ABF48477.1|	*Panax ginseng* dehydrin 4	953
contig04579	pop:POPTR_820375	Hypothetical protein	948
contig02872	sp|P04778|	Chlorophyll a-b binding protein 1	941
contig04578	emb|CAH59408.1|	Hypothetical protein	920
contig02875	sp|P27495|	Chlorophyll a-b binding protein 40	915
contig04448	sp|Q42580|	Peroxidase 21	911
contig02517	pop:POPTR_718004	Hypothetical protein	890
contig02408	ref|XP_002299525.1|	Hypothetical protein	887
contig02549	ref|YP_003934332.1|	Photosystem II PsbK protein	859
**Leaf**			
contig00303	rcu:RCOM_0030020	Ribulose bisphosphate carboxylase small chain	19345
contig00304	sp|P10795|	Ribulose bisphosphate carboxylase small chain 1A	18860
contig00004	rcu:RCOM_1571640	Ribulose bisphosphate carboxylase small chain 1B	11627
contig00270	rcu:RCOM_1158790	Ribulose bisphosphate carboxylase small chain	7879
contig00520	rcu:RCOM_1158790	Ribulose bisphosphate carboxylase small chain	6692
contig00325	sp|P23540|	Ribonuclease	3909
contig01392	sp|P49108|	Photosystem II 10 kDa polypeptide	3202
contig00936	sp|P27497|	Chlorophyll a-b binding protein M9	2041
contig00949	sp|P13851|	Chlorophyll a-b binding protein 1	1955
contig02266	ref|XP_002273037.1|	Hypothetical protein	1785
**Flower**			
contig01060	rcu:RCOM_1158790	Ribulose bisphosphate carboxylase small chain	999
contig04296	sp|P92919|	Chlorophyll a-b binding protein	942
contig01114	sp|Q40250|	Ribulose bisphosphate carboxylase small chain	932
contig26298	zma:100192931	Histone H4	919
contig04288	sp|P07371|	Chlorophyll a-b binding protein AB80	887
contig05604	ref|XP_002312129.1|	Hypothetical protein	881
contig33033	pop:POPTR_826664	Hypothetical protein	830
contig00507	gb|AAX40470.1|	Hypothetical protein	788
contig04848	sp|Q40519|	Photosystem II 10 kDa polypeptide	772
contig14522	sp|P50156|	Probable aquaporin TIP1-1	754

In the stem-derived cDNA library, the most highly expressed gene encoded phloem protein 2 (PP2)-like protein. PP2 is involved in the assimilate stream and has the capacity to interact with the mesophyll plasmodesmata to increase their size exclusion limit as well as cell-to-cell trafficking [24]. The second most highly expressed gene encodes a dehydration-related protein involved in the response to environmental stresses [25]. Genes encoding photosynthesis-related proteins, such as chlorophyll a-b binding proteins, peroxidase and the photosystem II PsbK protein, were also found to be abundant in the stem. Genes encoding chlorophyll a-b binding proteins were the most highly expressed genes in the stem-, leaf- and flower-derived libraries, and the root-derived cDNA libraries also contained several transcripts of chlorophyll a-b binding genes. This difference may be because stems, leaves and flowers are all chloroplast-containing tissues, whereas there are no chloroplasts in the tissues found in the root.

The most highly expressed genes in the leaf-derived cDNA library encoded proteins including ribulose bisphosphate carboxylase, photosystem II 10 kDa polypeptide and the chlorophyll a-b binding protein, as well as some proteins with an undefined function. Ribulose biphosphate carboxylase was also the most abundant transcript in the flower-derived libraries. Ribulose bisphosphate carboxylase catalyzes the initial step in the photosynthetic fixation of carbon dioxide [26].

Histone H4, aquaporin and a photosystem II 10 kDa polypeptide represented the three dominant transcripts in the flower-derived cDNA library. Histone H4 acetylation within euchromatic and heterochromatic domains plays a key role in DNA replication [27]. In higher plants, aquaporins are water channel proteins that facilitate the passage of water through biological membranes and play a crucial role in plant growth [28].

All of the predominantly expressed transcripts in each of these four tissue-derived cDNA libraries belong to the group of 50,957 genes shared by the four tissues. These genes are normally associated with housekeeping functions and play key roles in *P. ginseng* growth and development. Some housekeeping genes exhibit high expression levels in specific tissues, as observed for transport-related genes in the stem and photosynthesis-related genes in the leaf. This phenomenon can be explained by the fact that the proteins encoded by these genes are responsible for the characteristic functions of the corresponding tissues.

### Identification and characterization of potential miRNAs in *P. ginseng*

miRNAs are a class of noncoding, small endogenous RNAs, ~22 nucleotides (nt) in length that have been shown to regulate gene expression at the post-transcriptional level by targeting mRNAs for degradation or inhibiting protein translation [29]. There are currently 4,743 miRNAs that have been identified from 51 plant species deposited in the miRBase database [30]. However, no miRNAs have been identified in *P. ginseng* until now. Because only mature miRNA sequences (rather than precursor sequences) are conserved among plant species, mature miRNA sequences were used as queries for BLAST searches against the high-quality *P. ginseng* reads derived from our experiments. This process yielded 8,375 reads that were found to significantly match at least one mature miRNA sequence with no more than two mismatches and that could be related to either a target or an miRNA precursor sequence. A total of 3,707 noncoding reads were obtained after removing repeat and protein-coding sequences. Ultimately, we identified 14 candidate *P. ginseng* miRNAs with a proper miRNA precursor secondary structure and a minimal folding free energy index (MFEI) of at least 0.85 (Table 4).

**Table 4:** **Candidate miRNAs in *P. ginseng***

**miRNA**	**Query miRNAs**	**ML**	**Mature sequence**	**NM**	**Arm**	**GC%**	**PL**	**MFE**	**MFEI**	**EST ID**
pgi-miR1128	ssp-miR1128	21	UGCUACUCCCUCCGUCCCAAA	5	5^′^	22	290	99.2	1.55	G8WERZI02JSLMP
pgi-miR827	tcc-miR827	21	UUAGAUGAUCAUCAGCAAACA	1	3^′^	32	117	43.2	1.15	G8WERZI01D2S8J
pgi-miR1439	osa-miR1439	21	UUUUGGAACGGAGGGAGUAUU	4	3^′^	28	294	92.6	1.12	G8WERZI01A17SA
pgi-miR5658	ath-miR5658	21	AUGAUGAUGAUGAUGAUGAGG	3	3^′^	39	80	34.7	1.11	G9IMXQ301ESZFB
pgi-miR396i-3p	gma-miR396i-3p	21	GUUCAAUAAAGCUGUGGGAAG	1	3^′^	43	119	54.6	1.07	G9IMXQ302GOGE4
pgi-miR390b	bdi-miR390b	21	AAGCUCAGGAGGGAUAGCGCC	1	5^′^	39	175	72.5	1.06	G9IMXQ302H2ZM1
pgi-miR5021	ath-miR5021	20	GAAGAAGAAGAAGAAGAAAA	3	5^′^	32	134	44.8	1.04	G9IMXQ301DYCTK
pgi-miR156b	vun-miR156b	21	UGACAGAAGACUAGAGAGCAC	1	5^′^	44	112	49.6	1.01	G9IMXQ302IGRXR
pgi-miR403b	tcc-miR403b	21	UUAGAUUCACGCACAAACUCG	3	3^′^	41	117	46.8	0.98	G9IMXQ301CU7GZ
pgi-miR172	aau-miR172	22	UGAGAAUCUUGAUGAUGCUGCA	3	3^′^	44	171	71	0.94	G9IMXQ301B4R7M
pgi-miR408	vun-miR408	21	AUGCACUGCCUCUUCCCUGGC	4	3^′^	50	115	53.6	0.93	G9IMXQ301BH247
pgi-miR399d	tcc-miR399d	21	UGCCAAAGGAGAUUUGCCCGG	3	3^′^	41	122	45.2	0.9	G8WERZI01A00R5
pgi-miR482	gra-miR482	22	UCUUGCCAAUUCCUCCCAUUCC	3	3^′^	43	97	37.2	0.89	G9IMXQ301C4CDG
pgi-miR3441.1	aly-miR3441.1	20	UUCAAAGCCUCUUUGAAGGA	5	3^′^	38	68	21.9	0.85	G9IMXQ301DXOD1

*ML*, mature length; *NM*, number of mismatches in miRNA/miRNA* duplexes; Arm, location of mature miRNA; *PL*, precursor length; *MFE*, minimal folding free energy; *MFEI*, minimal folding free energy index; *EST ID*, the high quality reads number which can be accessed in the SRA Sequence Database under project accession number SRP015263.

Mature miRNA sequences can be located on either arm of the secondary stem-loop hairpin structure of the potential miRNA precursor (pre-miRNA). Of the 14 identified *P. ginseng* miRNAs, 4 were found to be located on the 5^′^ arm of the stem-loop hairpin structure, while 10 resided on the 3^′^ arm. The length of the putative *P. ginseng* miRNAs varied from 20 to 22 nt, with an average of 21 ± 0.5 nt. The majority (10 out of 14, or 71.4%) of the miRNAs were 21 nt in length. The length of the *P. ginseng* pre-miRNAs varied from 68 to 294 nt, averaging 143 ± 67 nt. The length distribution of the miRNAs and their precursor sequences was similar to the distributions described in previous reports in other plant species [12,31].

The minimal folding free energy (MFE) is important for the formation of RNA secondary structures. Generally, the lower the MFE, the more stable the secondary structure of a given RNA sequences. The average MFE value obtained in the present study for the *P. ginseng* miRNAs was −54.78 ± 21.82 kcal/mol, with a range of −21.9 to −99.2 kcal/mol. The minimal folding free energy index (MFEI) is a criterion for distinguishing miRNAs from other RNAs. Previous studies have shown that a sequence is more likely to be a potential miRNA if it presents an MFEI value greater than 0.85 [32]. For the 14 newly identified *P. ginseng* miRNAs, the average MFEI was 1.04 ± 0.17, with a range of 0.85 to 1.55. The secondary structures of the putative *P. ginseng* miRNA precursors are reported in Additional file 6.

### Target prediction for the *P. ginseng* miRNAs

Identification of miRNA target genes, in addition to constituting indirect existence evidence of miRNAs, is a fundamental step for the determination of biological function for miRNAs. Evolutionarily conserved targets have been shown to be useful in testing the effectiveness of miRNA target detection. A perfect, or near perfect, complementarity between an miRNA and its target mRNA, which is a peculiar feature of plant miRNAs, provides a powerful tool for the identification of target genes through BLAST analysis of mature miRNA sequences against EST sequences. After carefully considering the alignment results, we predicted at least one target for 7 miRNAs and a total of 100 potential targets for 14 miRNAs (Additional file 7). There were 37 and 51 targets predicted for miR5658 and miR5021, respectively, while the targets associated with other miRNAs were much less abundant, or may have failed to be sequenced. Zhou et al. detected a large number of targets for miR156 and miR396 and a small number for miR162, miR167, miR395, miR398 and miR399 in rice [33]. miRNAs with a large number of targets may represent nodes in gene regulatory networks, while those with a small number of targets may act through more specialized pathways.

Several studies have demonstrated that miRNAs can target transcription factors that control plant growth and development [13,31]. In the present study, the putative *P. ginseng* miR172 was predicted to target the transcription factor APETALA2 (AP2), which plays an important role in the control of the flowering time and floral morphology. miR172 has also been shown to target AP2 in tobacco, the opium poppy and *Brassica oleracea*[34-36]. Aukerman and Sakai found that overexpression of miR172, which targets AP2, causes early flowering and suppresses the floral organ specification in *Arabidopsis*[37]. In addition, this study suggested that MYB proteins might be the target of miR5021 in *P. ginseng*. Previous studies have also shown that MYB proteins may be targeted by miR5021 in *B. oleracea*[36]. The MYB transcription factors found in plants constitute a superfamily of proteins with a conserved MYB DNA binding domain that play a regulatory role in developmental processes and defense responses [38]. Zinc finger protein family members were predicted to be targeted by miR1128, miR5658 and miR5021. Zinc-finger proteins orchestrate the responses of plants to changes in environmental conditions and play a central role in reactive oxygen and abiotic stress signaling in *Arabidopsis*[39]. Other miRNAs identified in this study, such as miR403b, miR172, miR3441.1 and miR1439, can be considered putative regulators of gene expression at the protein level.

### Identification of simple sequence repeats (SSRs) in *P. ginseng*

SSRs are one of the most powerful types of molecular marker because of their relative abundance and ease of generation, and they have been widely applied for molecular-assisted selection (MAS) in plant breeding programs [40]. SSR markers derived from expressed sequence tags are likely to be even more transferable across lines, populations and species than random genomic SSRs [41]. In this study, a total of 13,044 SSRs were identified from 178,145 unigenes, with 1,582 of the *P. ginseng* unigenes containing at least two SSRs. The observed frequency of unigenes was 7.3%, and the distribution density was 172.5 per Mb. As is shown in Table 5, the most abundant repeat type was dinucleotides (51.0%, 6,659), and the most common number of tandem repeats was 6 (24.4%, 3,179). The dominant repeat motif was AG/CT, with a frequency of 24.5% (Additional file 8). The primer pairs for each SSR conforming to a series of primer-designing parameters (see Methods) are offered in Additional file 9 for further investigation of the potential of these SSRs as genetic markers.

**Table 5:** **Frequencies of repeat types with repeat numbers in *P. ginseng *EST-SSRs**

**Motif length**	**Repeat number**								**Total**	**%**
	**4**	**5**	**6**	**7**	**8**	**9**	**10**	**>10**		
Di	-	-	2,169	1,530	999	631	414	916	6,659	51.0
Tri	-	1,868	786	408	226	118	67	162	3,635	27.9
Tetra	990	255	116	57	22	7	1	7	1,455	11.2
Penta	358	116	40	15	1	1	1	0	532	4.1
Hexa	442	187	68	34	15	5	6	6	763	5.8
total	1,790	2,426	3,179	2,044	1,263	762	489	1,091	13,044	-
%	13.7	18.6	24.4	15.7	9.7	5.8	3.7	8.4	-	-

## Conclusions

In this study, a large-scale EST investigation was performed in root, stem, leaf and flower tissues from *P. ginseng*. The obtained EST dataset provides a comprehensive resource for gene discovery and genetic analyses in *P. ginseng*. The genes identified in this study will help to decipher the molecular mechanisms of secondary metabolism in *P. ginseng*. Moreover, this study identified putative miRNAs from *P. ginseng* and their targets, thus representing a foundation for further research into transcriptional regulation. Finally, the large set of SSRs identified in this work provides abundant genetic markers for molecular breeding and genetic applications in this species.

## Methods

### Plant material

Actively growing 4-year-old ginseng (*P. ginseng* C. A. Meyer) was harvested from a field plot in Kuandian County, Liaoning Province, China, on June 27, 2011. At that time, the temperature ranged from 14.3 ~ 24.7°C, averaging 19.4°C. The four seasons in Kuandian County are distinct. A majority of the annual rainfall occurs in July and August. The monthly 24-hour average temperatures range from −11.5°C in January to 22.5°C in July, while the annual mean is 6.5°C. The average relative humidity is 70%, and the frost-free period is 140 days. Main roots, stems, leaves and flower buds were collected separately from a single plant and cut into small pieces followed immediately by storage in liquid nitrogen until further processing.

### RNA preparation

Total RNA was isolated from roots, stems, leaves and flowers using the RNeasy Plus Mini kit (Qiagen, Valencia, CA, USA). Quality control was performed in the samples using RNA 6000 Nano LabChips with Bioanalyzer 2100 (Agilent Technologies, PaloAlto, CA, USA), and the obtained concentrations were assessed using a NanoDrop ND-1000 spectrophotometer (Nano-Drop Technologies, Wilmington, DE, USA) before processing. The RNA samples were treated with TURBO DNase (Ambion, Austin, TX, USA) at a concentration of 1.5 units/μg of total RNA prior to cDNA synthesis.

### cDNA synthesis and sequencing

Four cDNA libraries were constructed from the roots, stems, leaves and flowers of *P. ginseng*. First-strand cDNA was produced from 2 μg of total RNA extracted from each of the *P. ginseng* tissues using the SMART cDNA synthesis kit (Clontech, Palo Alto, CA, USA) according to the manufacturer’s instructions, with slight modifications, as described in a previous report [21]. For double-stranded cDNA (ds cDNA) synthesis, the cDNA was amplified using PCR Advantage II polymerase (Clontech, Palo Alto, CA, USA) with the following thermal profile: 1 min at 95°C, followed by 19 cycles of 95°C for 15 s, 65°C for 30 s and 68°C for 6 min. Then, 5 μl of the obtained PCR products were electrophoresed in a 1% agarose gel to determine the amplification efficiency. Finally, all of samples were amplified over 12 cycles. Approximately 13 μg of the amplified ds cDNA was purified using the Pure-Link™ PCR purification kit (Invitrogen Life Technologies Corp, Carlsberg, Calif, USA) and the cDNA was subsequently treated with *Bsg*I (NEB, Ipswich, MA, USA) overnight and recovered using the QIAquick PCR Purification Kit (Qiagen, Valencia, CA, USA).

Next, 500 ng of ds cDNA from each tissue was used for shotgun cDNA library construction according to the manual of the GS FLX Titanium Rapid Library Preparation Kit (454 Life Sciences Corp, Branford, CT, USA). The DNA was nebulized for 1 minute and then end-repaired using T4 DNA polymerase and T4 polynucleotide kinase. Adaptors were blunt-end ligated to the fragment ends using T4 DNA ligase. AMPure beads (Agencourt Bioscience Corp, Beverly, MA, USA) were employed to remove small DNA fragments and to collect DNA fragments between 600 bp and 900 bp in length.

Using emulsion PCR, the DNA molecules in the shotgun library were amplified with the GS FLX Titanium LV emPCR package (454 Life Sciences Corp, Branford, CT, USA), according to the manufacturer’s recommendations. After amplification, the beads bound to amplified molecules were collected, and the emulsion oil was removed via washing, according to the manufacturer’s protocol. Beads bound to a sufficient number of copies of the clonally amplified library fragments were selected using a specified enrichment procedure and were subsequently counted with a Multisizer 3 Coulter Counter (Beckman Coulter, Fullerton, CA, USA) prior to sequencing.

Following emulsion PCR enrichment, the selected beads were loaded into the wells of a Titanium Series PicoTiterPlate device via centrifugation. Then, 454 sequencing was performed according to the manufacturer’s instruction manual (454 Life Sciences Corp, Branford, CT, USA). Image analysis, signal processing, and base calling were conducted using Newbler 2.3 software (454 Life Sciences Corp, Branford, CT, USA).

### EST assembly and data analysis

The raw 454 reads were screened and trimmed for weak signals using GS FLX pyrosequencing software to yield high-quality reads. The primer and adapter sequences were trimmed from the high-quality sequences to obtain clean ESTs. Then, sequences shorter than 50 bp were removed. Finally, the data from the 454 reads was assembled into unigenes (containing contigs and singletons) using GS De NovoAssembler software, v2.6 (454 Life Sciences Corp, Branford, CT, USA). Functional annotation was carried out against a series of nucleotide and protein databases, including the Swiss-Prot [42], InterPro [43], Kegg [44], Nr [45] and Nt [46] databases, using BLAST (version 2.2.17) with a common significance threshold cutoff of *E*-value ≤ 1e-5. The functional categories of these unique sequences were further analyzed according to GO terms based on InterPro GO slim provided by InterPro [43].

### Homology-based searches for miRNAs and target prediction

A set of known miRNAs was downloaded from miRBase (version 18.0, November 2011), comprising a total of 4,743 mature miRNA sequences from 51 plant species [30]. A homology-based search for miRNAs in *P. ginseng* was carried out using previously reported methods [12,31]. Subsequently, miRNA targets were predicted according to the method described in a previous report [31].

### SSRs detection and primer design

To detect SSRs in *P. ginseng*, we performed SSR analysis of the obtained unigenes using the microsatellite identification tool MISA [47]. The parameters were designed for identifying di-, tri-, tetra-, penta- and hexa-nucleotide motifs with a minimum of 6, 5, 4, 4 and 4 repeats, respectively [48]. Primer3 software was employed to design flanking primers for detecting microsatellites [49]. The main primer design parameters were set as follows: PCR products ranging from 100 to 300 nt, primer lengths ranging from 18 to 24 nt (with an optimum of 20 nt), a 60°C optimal annealing temperature, and a GC content from 40% to 65% (with an optimum of 50%) [48].

## Abbreviations

P. ginseng: *Panax ginseng* C. A. Meyer; NGS: Next-generation sequencing; BLAST: Basic local alignment search tool; Nt: Nucleotide; Bp: Base pair; cDNA: Complementary DNA; AACT: Acetyl-CoA acetyltransferase; HMGS: HMG-CoA synthase; HMGR: HMG-CoA reductase; MVK: Mevalonate kinase; PMK: Phosphomevalonate kinase; MVD: Mevalonate diphosphate decarboxylase; GPS: Geranylgeranyl pyrophosphate synthase; IDI: Isopentenyl diphosphate isomerase; FPS: Farnesyl diphosphate synthase; SS: Squalene synthase; SE: Squalene epoxidase; AS: Amyrin synthase; DS: Dammarendiol synthase; UGT: UDP glycosyltransferase; CYP450: Cytochrome P450; EST: Expressed sequence tag; GO: Gene ontology; Nt: NCBI non-redundant nucleotide; Nr: NCBI non-redundant protein; KEGG: Kyoto encyclopedia of genes and genomes; NCBI: National center for biotechnology information; MFE: Minimal folding free energy; MFEI: Minimal folding free energy index; miRNA: microRNA; pre-miRNA: miRNA precursor; AP2: APETALA2.

## Competing interests

The authors declare that they have no competing interests.

## Authors’ contributions

CFL conceived the study, built the cDNA libraries, performed 454 sequencing, participated in the data analysis and drafted the manuscript. YJZ performed most of the data analysis. XG participated in 454 sequencing. CS contributed to designing the study. HML and JYS helped to conceive the study. YL participated in the data analysis. LZW participated in cDNA library construction and 454 sequencing. JQ performed the SSR analysis. SLC initiated the project, designed the study and participated in study coordination. All authors read and approved the final manuscript.

## Supplementary Material

Additional file 1 **Length distributions of reads.** TIFF document for the length distributions of *P. ginseng* four tissues reads. Click here for file

Additional file 2 **Length distributions of contigs.** TIFF document for the length distributions of *P. ginseng* four tissues contigs. Click here for file

Additional file 3 **Summary of the 454 sequencing and assembly for *P. ginseng *four tissues.** DOCX document for the summary of 454 sequencing and assembly data. Click here for file

Additional file 4 **Summary of annotation statistics against public databases for *P. ginseng *four tissues.** DOCX document for the summary of annotation results. Click here for file

Additional file 5 **Transcripts involved in ginsenoside skeleton biosynthesis in *P. ginseng *four tissues.** DOCX document for the number of ginsenoside skeleton biosynthesis genes in *P. ginseng* each tissue and four tissues. Click here for file

Additional file 6 **Secondary structures of the putative miRNA precursors.** DOCX document for the predicted secondary structures of the putative miRNA precursors. Click here for file

Additional file 7 **miRNA potential target genes.** DOCX document for the predicted miRNA target genes. Click here for file

Additional file 8 **Occurrence of SSRs in *P. ginseng *unigenes.** DOCX document for the summary of occurrence of SSRs in *P. ginseng* unigenes. Click here for file

Additional file 9 **Primer pairs for each SSR.** XLSX document for the primer pairs for each SSR. Click here for file
