# Supplementary figures and images for: Transcriptome analysis reveals ginsenosides biosynthetic genes, microRNAs and simple sequence repeats in Panax ginseng C. A. Meyer
Source: BMC Genomics. 2013 Apr 11;14:245. doi: 10.1186/1471-2164-14-245 (PMC3637502; doi:10.1186/1471-2164-14-245)

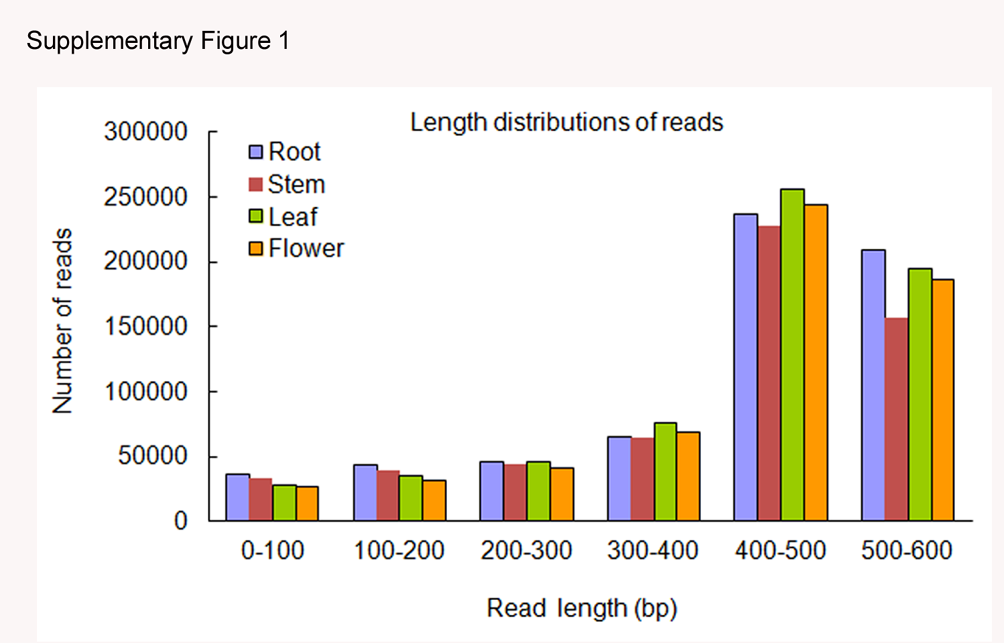

Supplement: Additional file 1 — Length distributions of reads. TIFF document for the length distributions of P. ginseng four tissues reads. [file 1471-2164-14-245-S1.tiff]

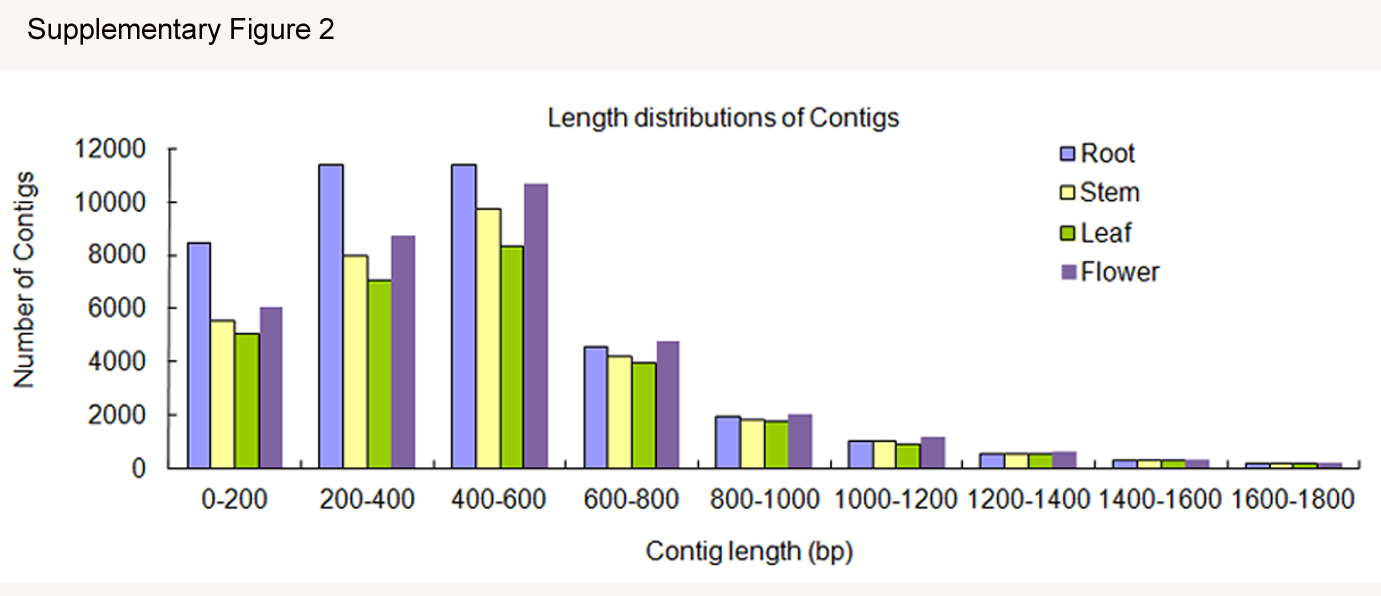

Supplement: Additional file 2 — Length distributions of contigs. TIFF document for the length distributions of P. ginseng four tissues contigs. [file 1471-2164-14-245-S2.tiff]
