# Supplementary material for: Transcriptome analysis reveals ginsenosides biosynthetic genes, microRNAs and simple sequence repeats in Panax ginseng C. A. Meyer
Source: BMC Genomics. 2013 Apr 11;14:245. doi: 10.1186/1471-2164-14-245 (PMC3637502; doi:10.1186/1471-2164-14-245)
Supplement: Additional file 3 — Summary of the 454 sequencing and assembly for P. ginseng four tissues. DOCX document for the summary of 454 sequencing and assembly data. [file 1471-2164-14-245-S3.docx]

**Table S1 - Summary of the 454 sequencing and assembly for *P. ginseng* four tissues**

|  | **Root** | | **Stem** | | **Leaf** | | **Flower** | |
| --- | --- | --- | --- | --- | --- | --- | --- | --- |
|  | No. of sequences | No. of bases | No. of sequences | No. of bases | No. of sequences | No. of bases | No. of sequences | No. of bases |
| **Sequencing** | | | | | | |  | |
| High-quality reads | 633,995 | 256,432,703 | 562,363 | 222,744,597 | 631,791 | 257,760,451 | 594,927 | 243,227,534 |
| Average high-quality read length (bp) | 404.5 |  | 396.1 |  | 407.9 |  | 408.8 |  |
| Reads used in assembly | 493,189 | 192,779,928 | 476,215 | 187,400,142 | 550,608 | 221,940,102 | 513,567 | 207,244,583 |
| **Contigs** | | | | | | |  | |
| Number of contigs ≥ 100 bp | 40,042 | 18,313,320 | 31,556 | 15,554,232 | 28,242 | 14,103,311 | 34,710 | 17,114,931 |
| Average length of contigs (bp) | 457.4 |  | 492.9 |  | 499.4 |  | 493.1 |  |
| Range of contig lengths (bp) | 100-15,455 |  | 100-8,017 |  | 100-6,206 |  | 100-5,664 |  |
| **Singletons** | | | | | | |  | |
| Number of singletons ≥ 100bp | 76,527 | 30,675,098 | 44,314 | 17,065,862 | 41,678 | 16,491,299 | 42,553 | 17,018,147 |
| Average length of singletons (bp) | 400.9 |  | 385.3 |  | 395.9 |  | 400.2 |  |
| Range of singleton lengths (bp) | 100-691 |  | 100-633 |  | 100-637 |  | 100-646 |  |
| **Unique sequences** | | | | | | |  | |
| Number of unigenes  (contigs and singletons) | 116,569 |  | 75,870 |  | 69,920 |  | 77,243 |  |
| Total coverage (bp) | 146,336,200 |  |  |  |  |  |  |  |
