# Supplementary material for: Transcriptome analysis reveals ginsenosides biosynthetic genes, microRNAs and simple sequence repeats in Panax ginseng C. A. Meyer
Source: BMC Genomics. 2013 Apr 11;14:245. doi: 10.1186/1471-2164-14-245 (PMC3637502; doi:10.1186/1471-2164-14-245)
Supplement: Additional file 4 — Summary of annotation statistics against public databases for P. ginseng four tissues. DOCX document for the summary of annotation results. [file 1471-2164-14-245-S4.docx]

| The public database | Root | | Stem | | Leaf | | Flower | |
| --- | --- | --- | --- | --- | --- | --- | --- | --- |
|  | No. of sequences | Annotation percentage (%) | No. of sequences | Annotation percentage (%) | No. of sequences | Annotation percentage (%) | No. of sequences | Annotation percentage (%) |
| SwissProt | 14,933 | 37.3 | 15,009 | 47.6 | 14,036 | 49.7 | 16,554 | 47.7 |
| KEGG | 22,721 | 56.7 | 21,466 | 68.0 | 19,717 | 69.8 | 23,908 | 68.9 |
| COG | 5,809 | 14.5 | 6,104 | 19.3 | 5,984 | 21.2 | 6,689 | 19.3 |
| Nr | 23,044 | 57.5 | 21,575 | 68.4 | 19,815 | 70.2 | 24,040 | 69.3 |
| Nt | 21,182 | 52.9 | 19,973 | 63.3 | 18,380 | 65.1 | 22,091 | 63.6 |
| Total annotated | 25,171 | 62.9 | 22,945 | 72.7 | 20,998 | 74.4 | 25,421 | 73.2 |
| Total unique sequences | 40,042 |  | 31,556 |  | 28,242 |  | 34,710 |  |

**Table S2 - Summary of annotation statistics against public databases for *P. ginseng* four tissues**
