# Supplementary material for: Transcriptome analysis reveals ginsenosides biosynthetic genes, microRNAs and simple sequence repeats in Panax ginseng C. A. Meyer
Source: BMC Genomics. 2013 Apr 11;14:245. doi: 10.1186/1471-2164-14-245 (PMC3637502; doi:10.1186/1471-2164-14-245)
Supplement: Additional file 5 — Transcripts involved in ginsenoside skeleton biosynthesis in P. ginseng four tissues. DOCX document for the number of ginsenoside skeleton biosynthesis genes in P. ginseng each tissue and four tissues. [file 1471-2164-14-245-S5.docx]

**Table S3 - Transcripts involved in ginsenoside skeleton biosynthesis in *P. ginseng* four tissues**

| **Gene name** | **EC number** | **Root unigene number** | **Stem unigene number** | **Leaf unigene number** | **Flower unigene number** | **Total unigene number** |
| --- | --- | --- | --- | --- | --- | --- |
| Acetyl-CoA acetyltransferase | 2.3.1.9 | 5 | 5 | 3 | 5 | 18 |
| HMG-CoA synthase | 2.3.1.10 | 1 | 2 | 2 | 3 | 8 |
| HMG-CoA reductase | 1.1.1.34 | 5 | 4 | 10 | 13 | 32 |
| Mevalonate kinase | 2.7.1.36 | 3 | 1 | 4 | 1 | 9 |
| Phosphomevalonate kinase | 2.7.4.2 | 8 | 1 | 2 | 5 | 16 |
| Mevalonate-5-diphosphate decarboxylase | 4.1.1.33 | 3 | 3 | 2 | 3 | 11 |
| Cycloartenol synthase | 5.4.99.8 | 0 | 2 | 4 | 6 | 12 |
| Isopentenyl-PP- isomerase | 5.3.3.2 | 1 | 3 | 5 | 6 | 15 |
| Geranyl diphosphate synthase | 2.5.1.29 | 2 | 9 | 21 | 13 | 45 |
| Farnesyl diphosphate synthase | 2.5.1.1 2.5.1.10 | 2 | 4 | 4 | 4 | 14 |
| Squalene synthase | 2.5.1.21 | 1 | 3 | 7 | 11 | 22 |
| Squalene epoxidase | 1.14.99.7 | 3 | 7 | 4 | 7 | 21 |
| Beta-amyrin synthase |  | 0 | 1 | 1 | 4 | 6 |
| Dammarenediol-II synthase |  | 1 | 5 | 5 | 5 | 16 |
