# Supplementary material for: Transcriptome analysis reveals ginsenosides biosynthetic genes, microRNAs and simple sequence repeats in Panax ginseng C. A. Meyer
Source: BMC Genomics. 2013 Apr 11;14:245. doi: 10.1186/1471-2164-14-245 (PMC3637502; doi:10.1186/1471-2164-14-245)
Supplement: Additional file 6 — Secondary structures of the putative miRNA precursors. DOCX document for the predicted secondary structures of the putative miRNA precursors. [file 1471-2164-14-245-S6.docx]

**Additional file 6 - Secondary structures of the putative miRNA precursors**

pgi-miR1128


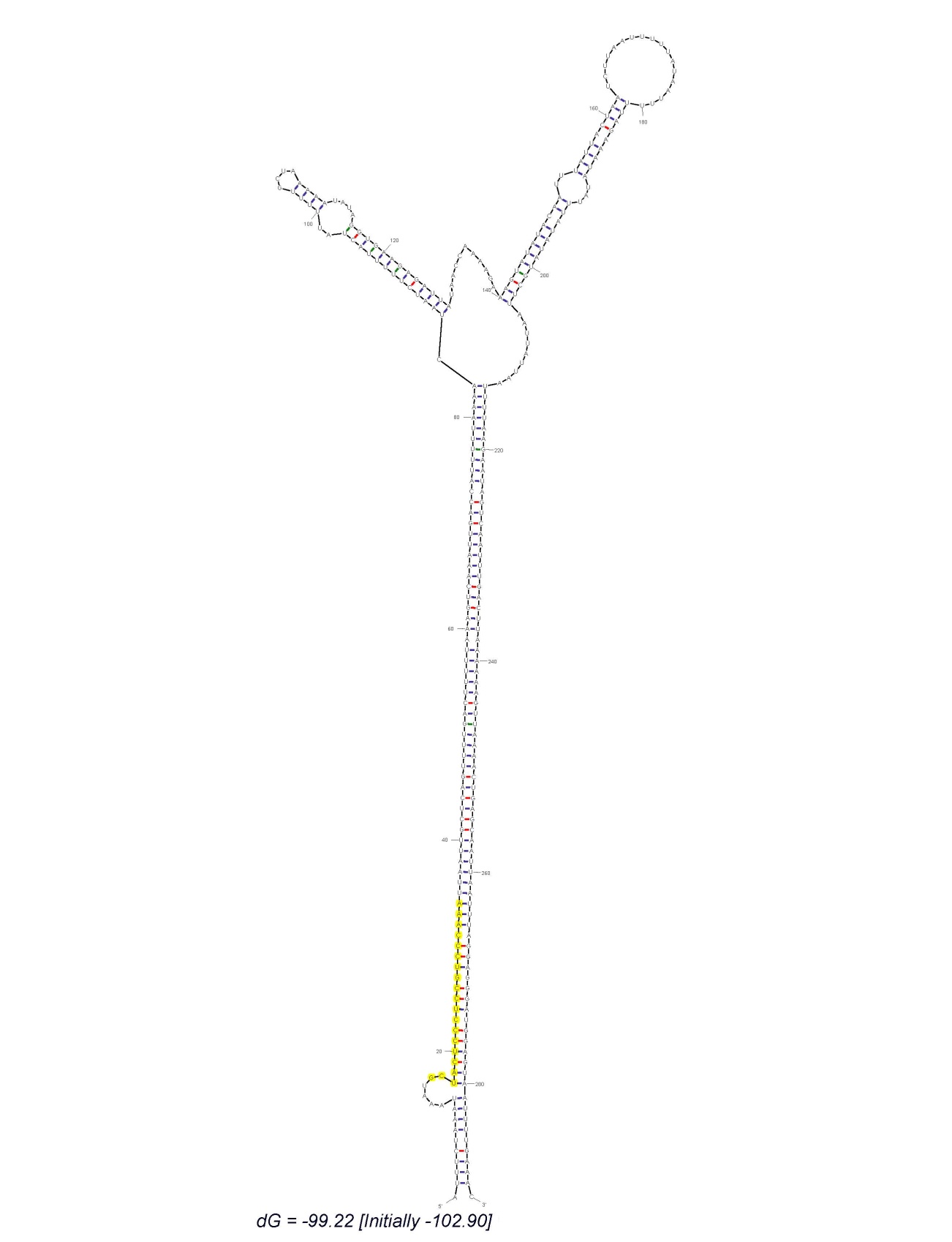


pgi-miR827
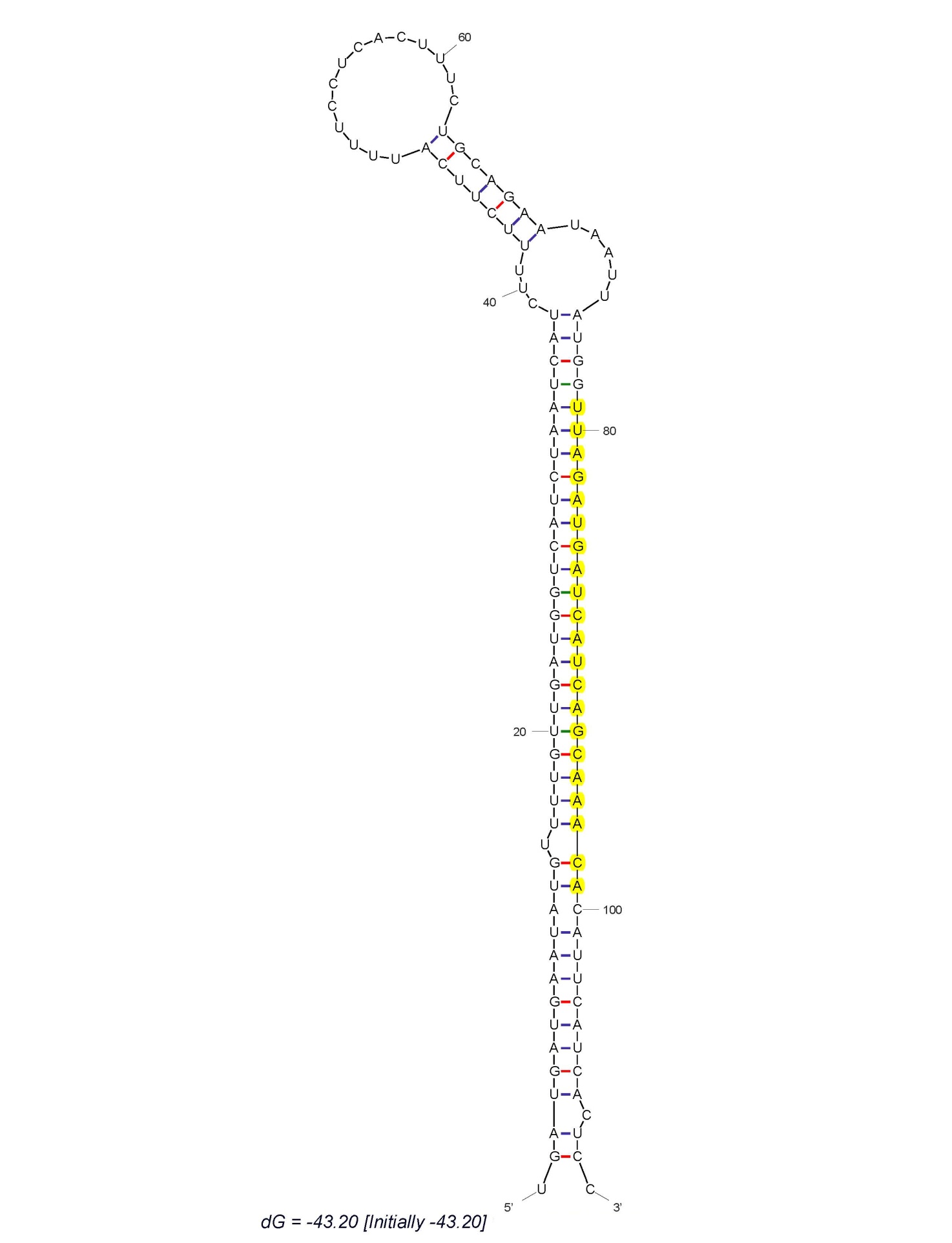


pgi-miR1439
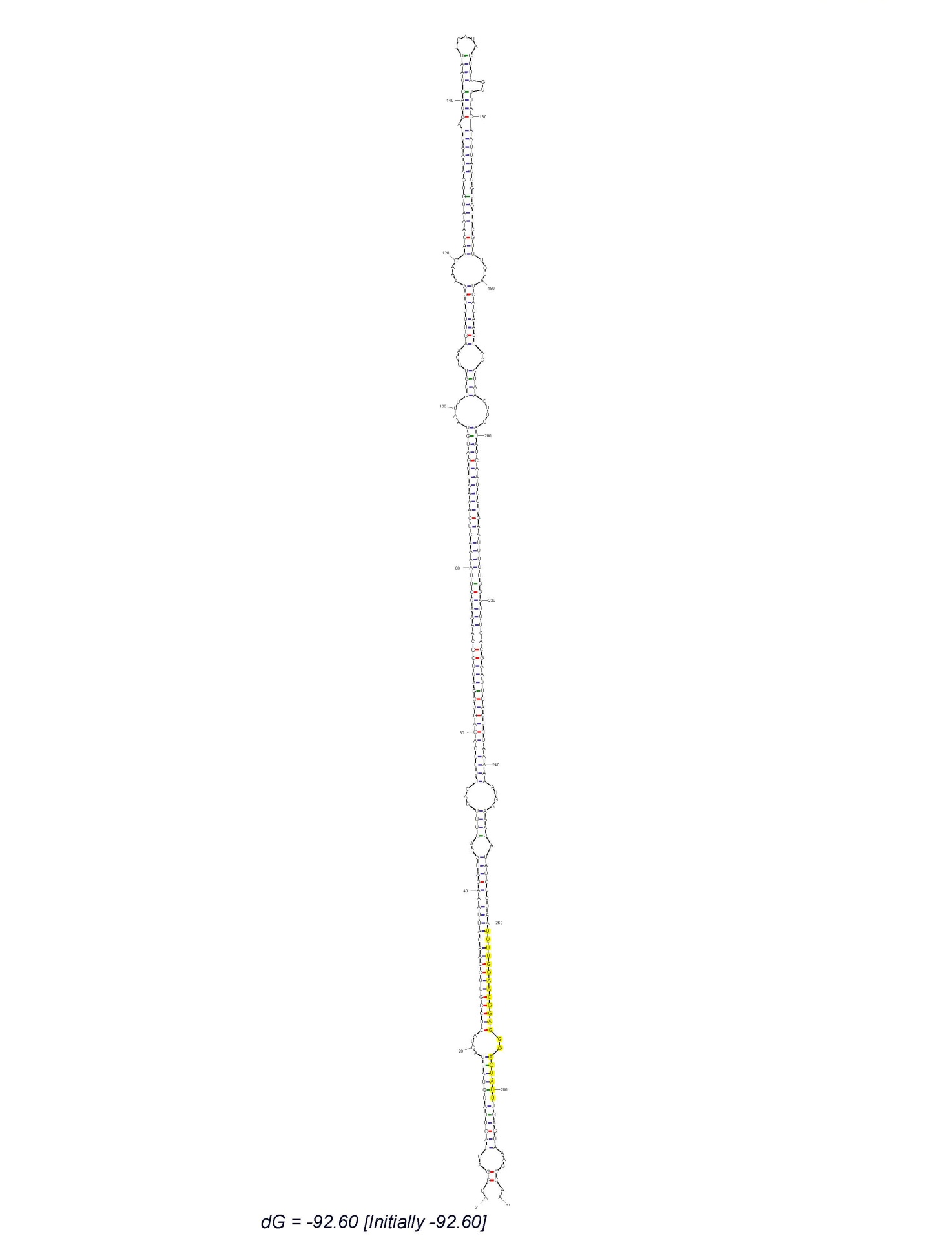


pgi-miR5658
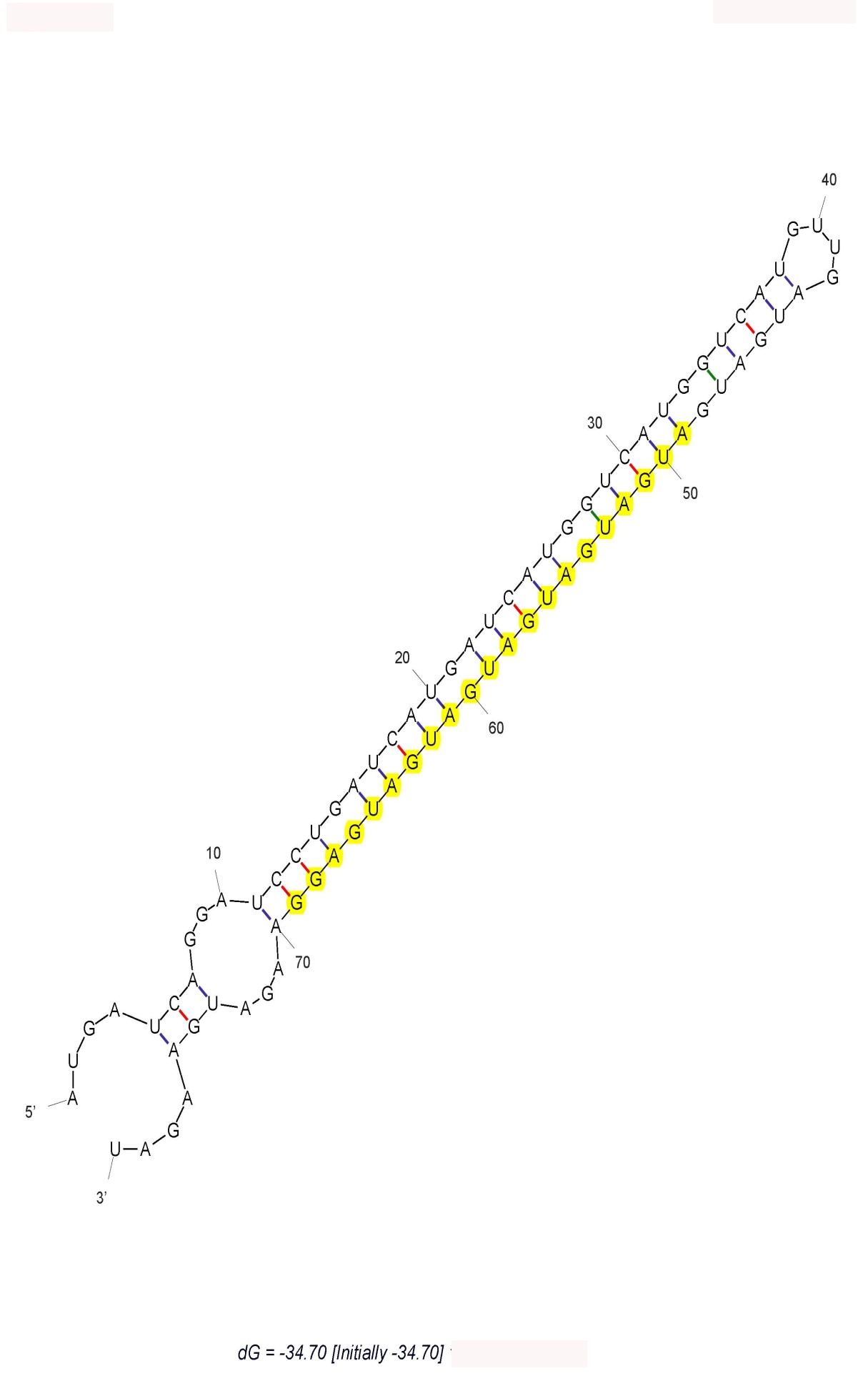


pgi-miR396i-3p


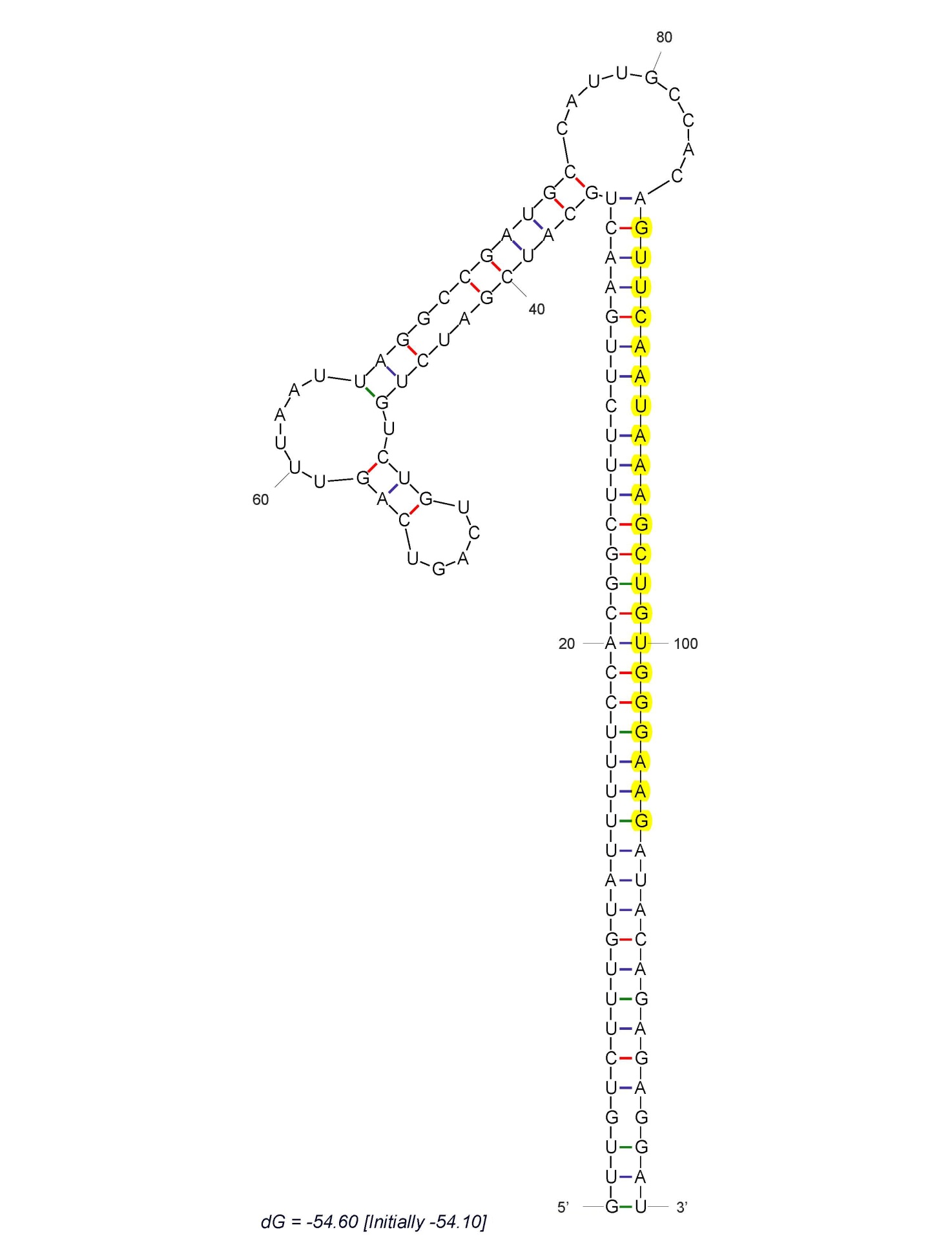


pgi-miR390b
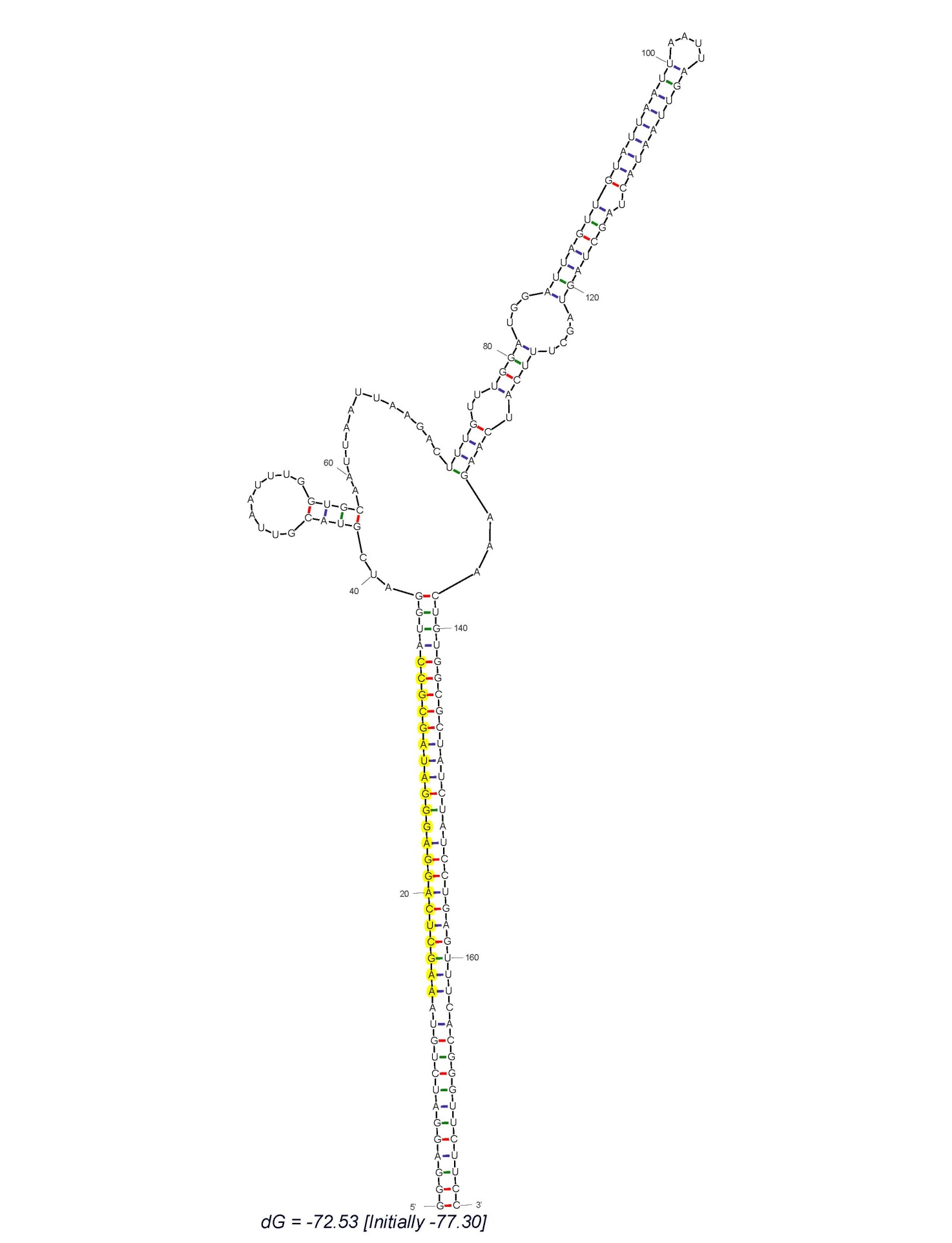


pgi-miR5021


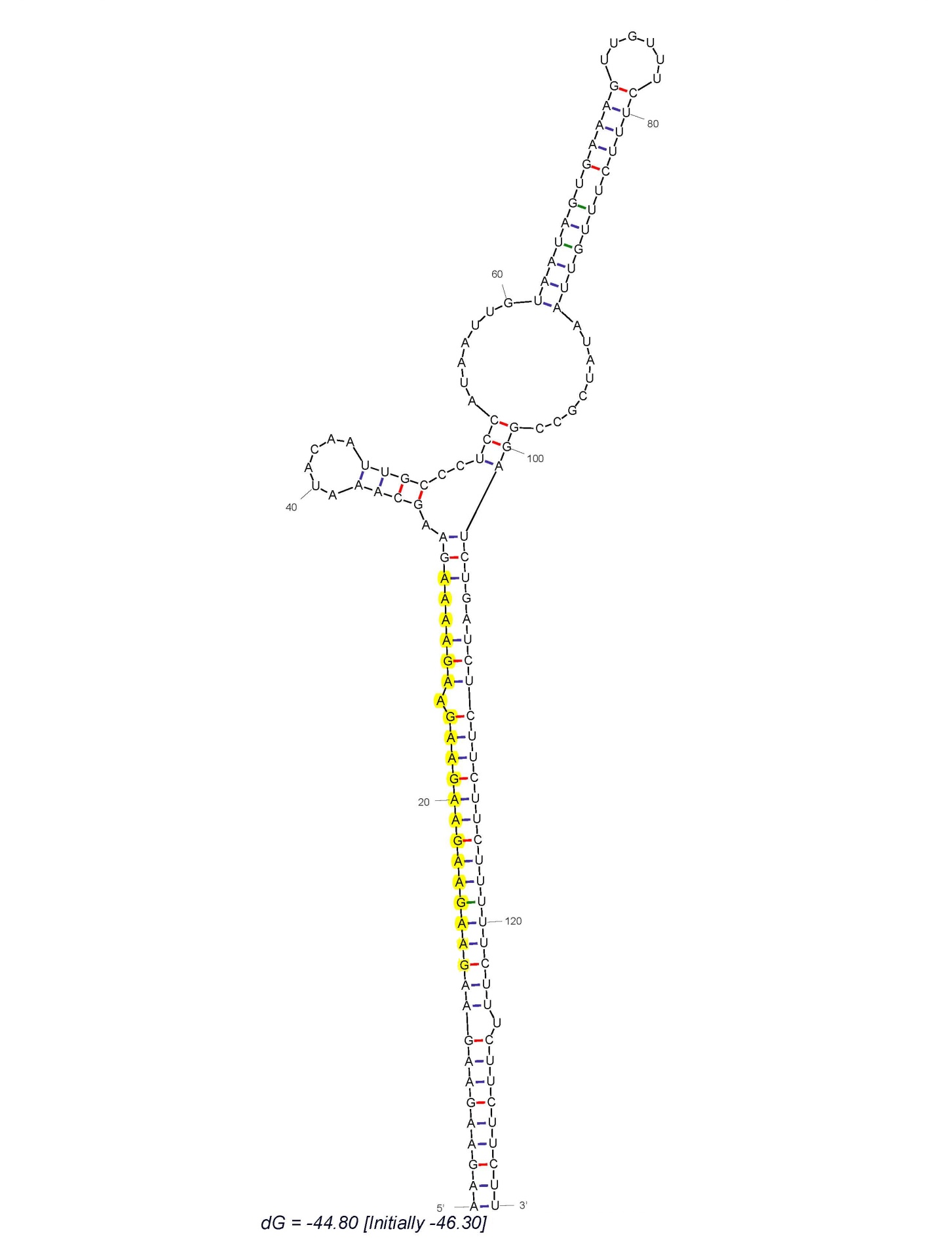


pgi-miR156b
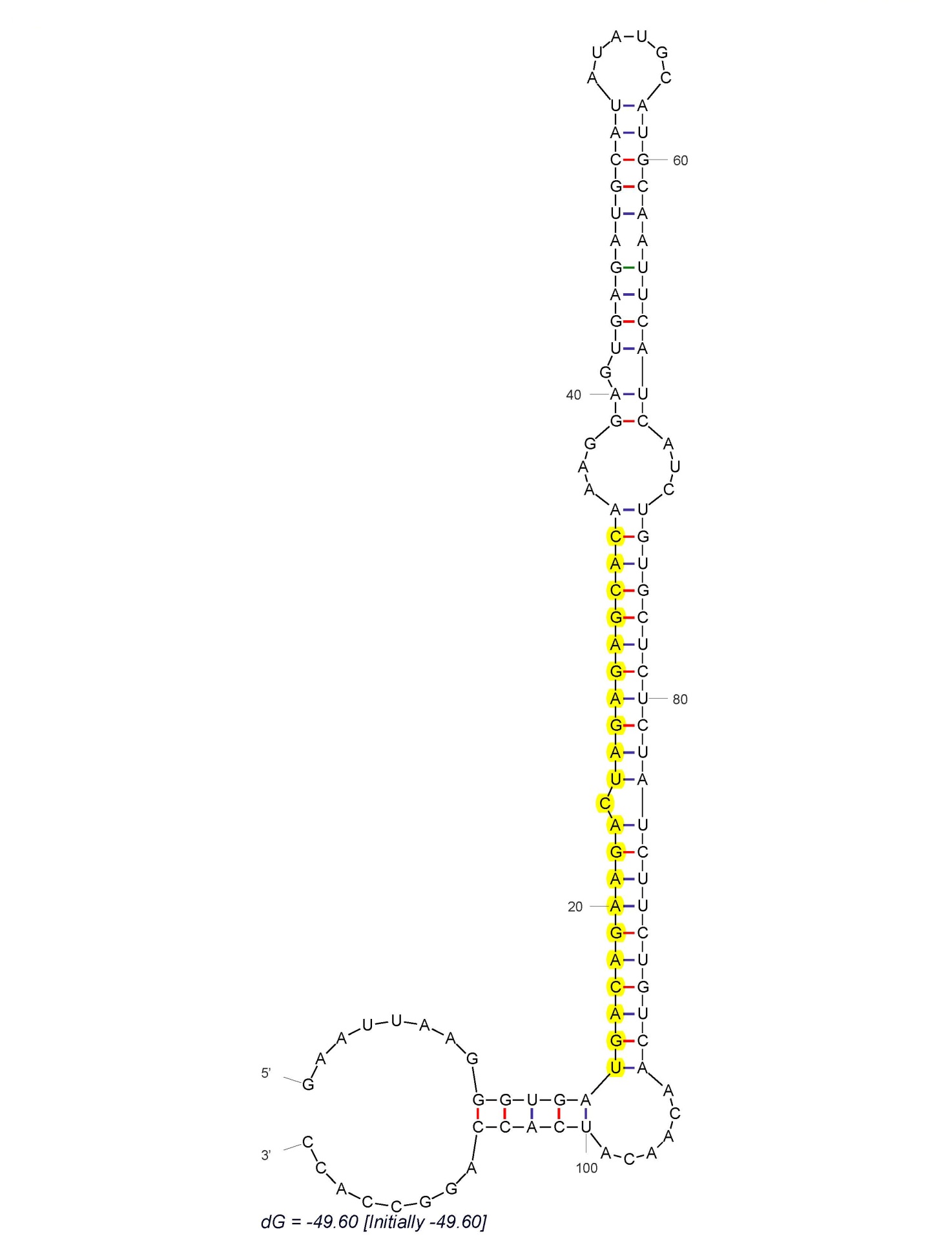


pgi-miR403b
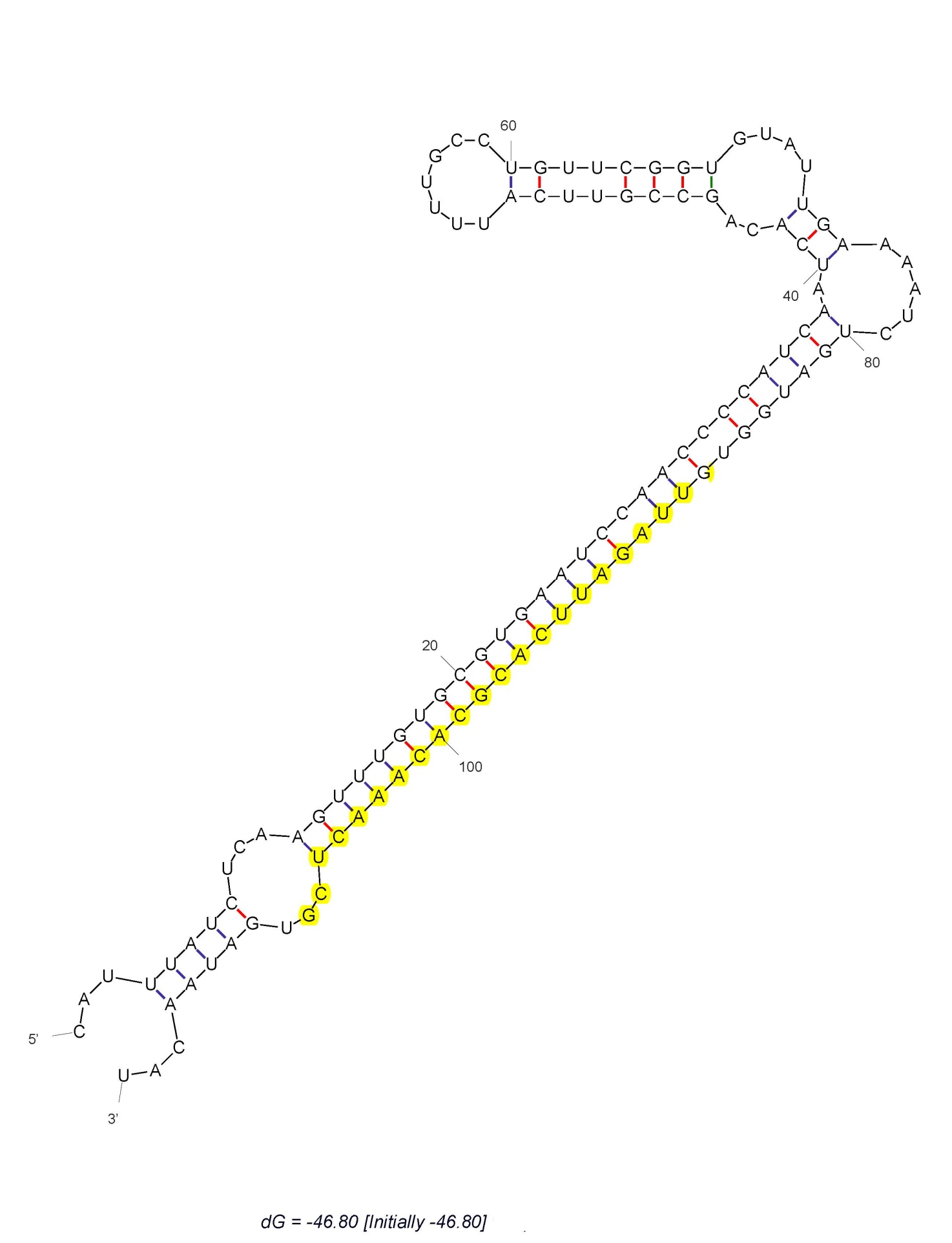


pgi-miR172
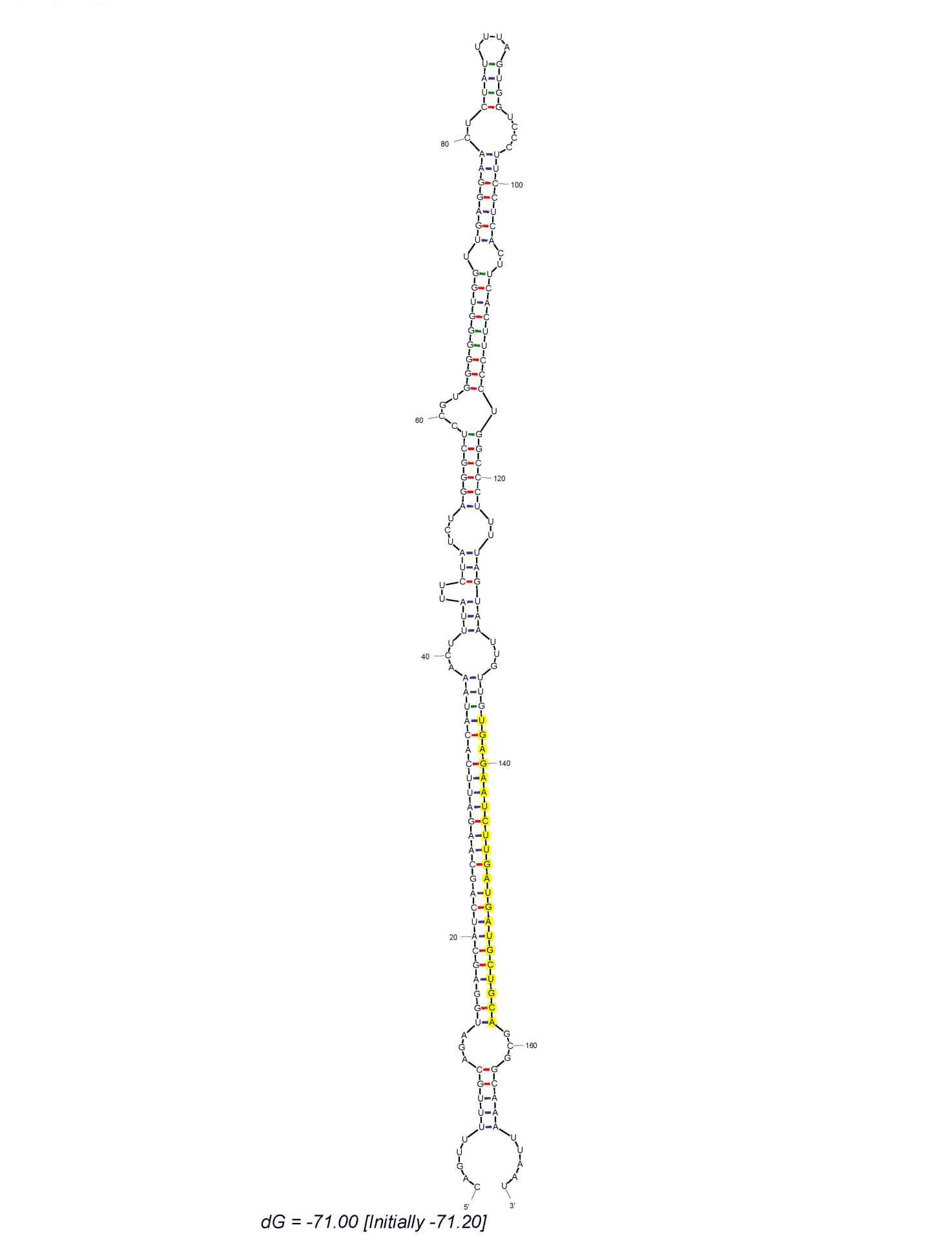


pgi-miR408
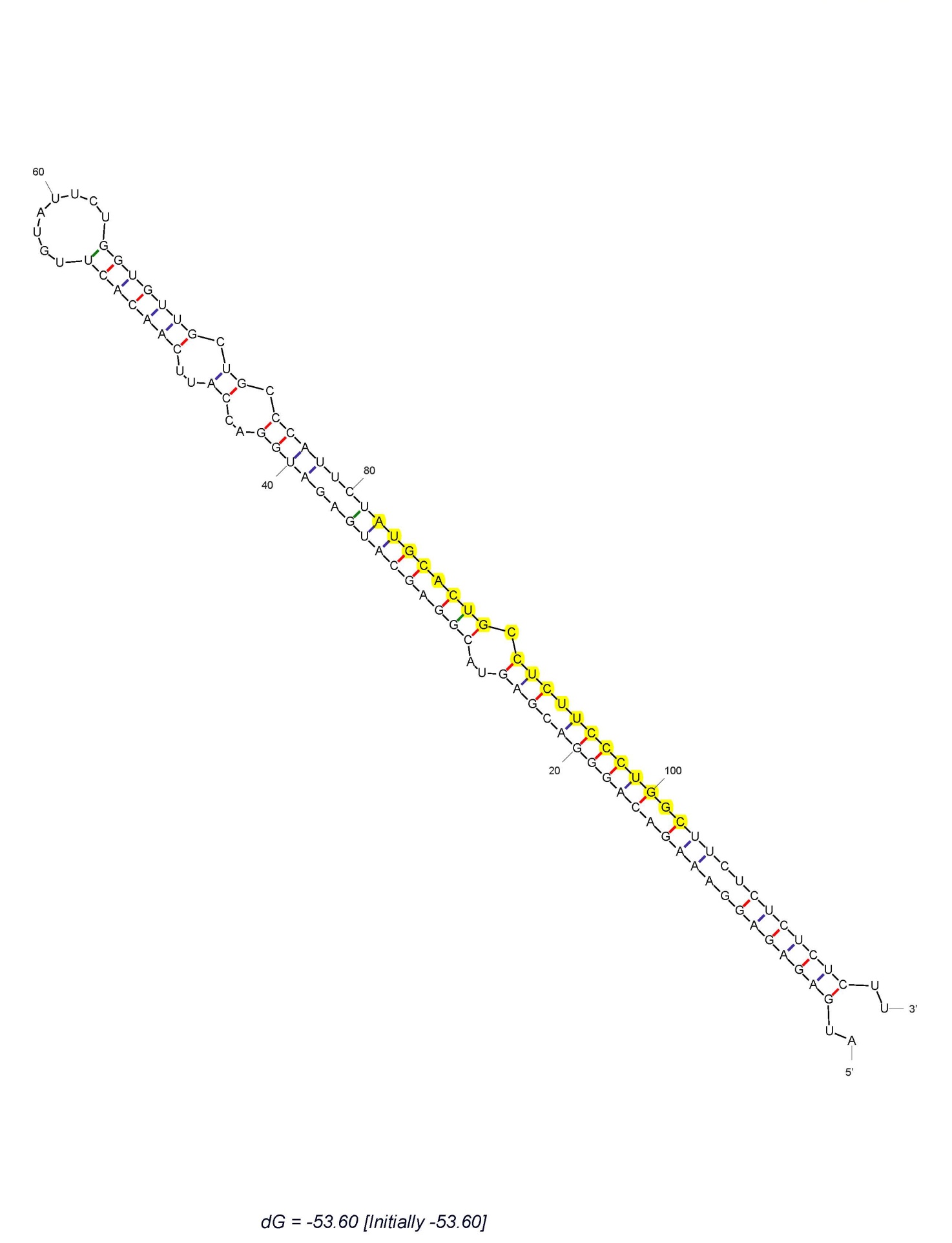


pgi-miR399d
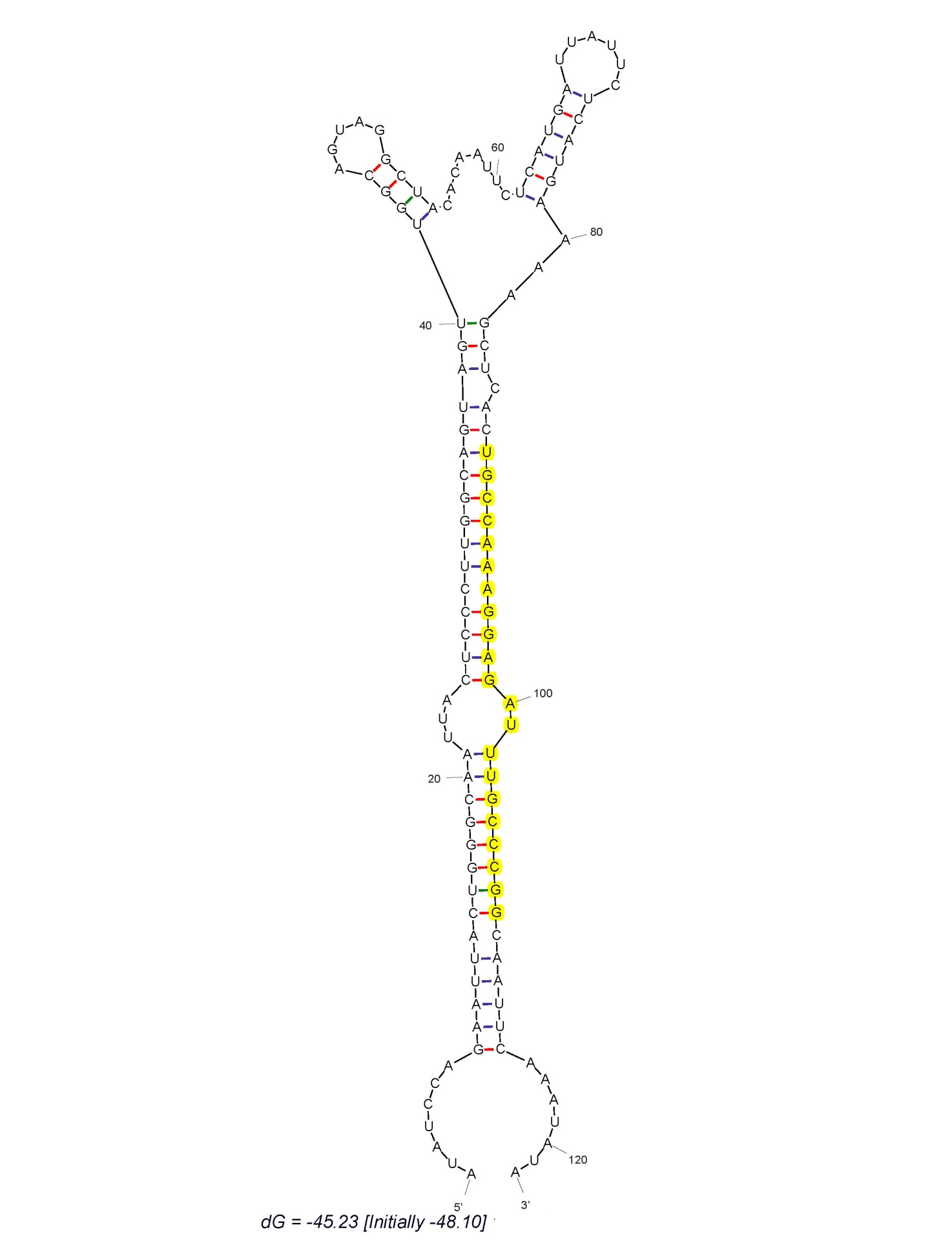


pgi-miR482
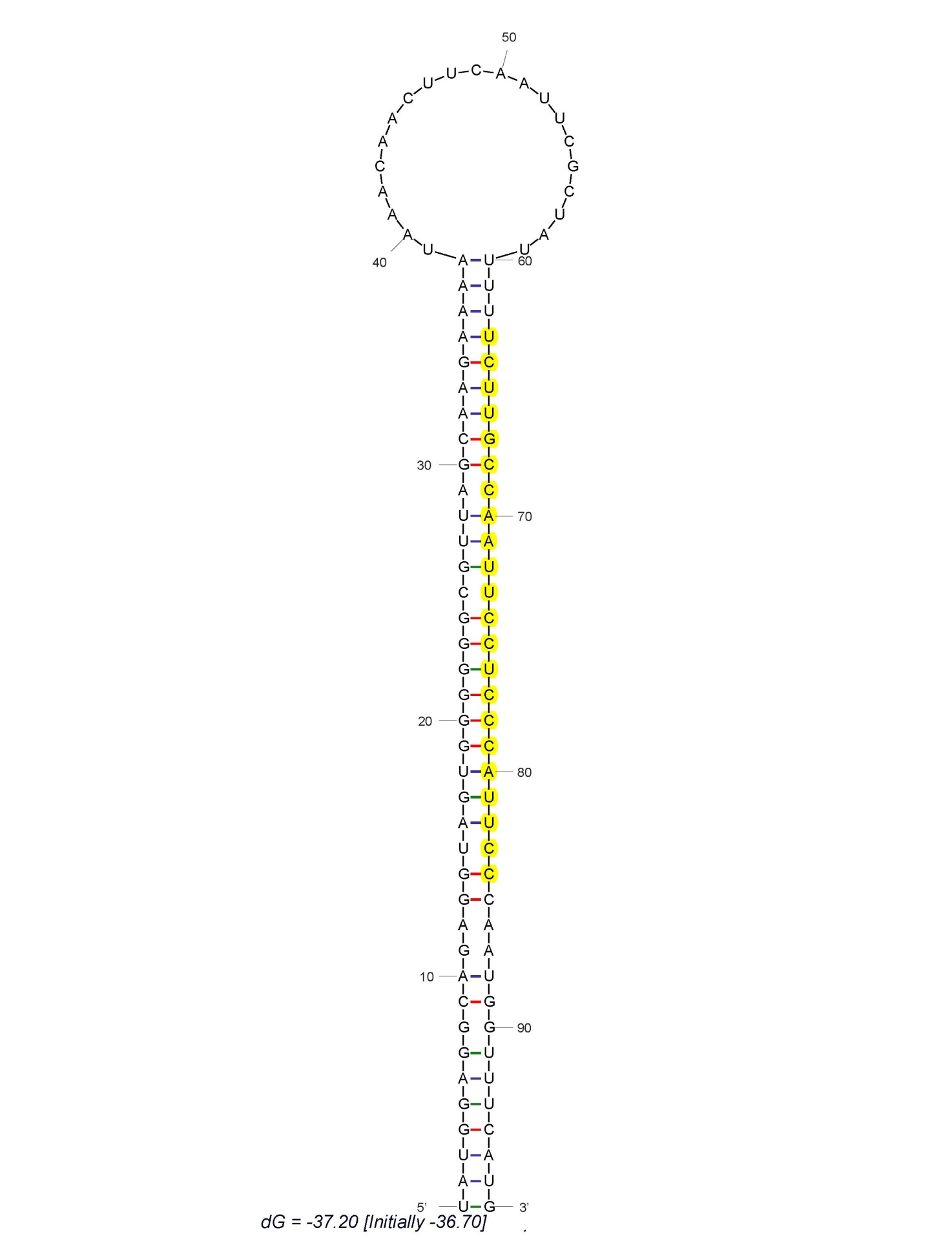


pgi-miR3441.1
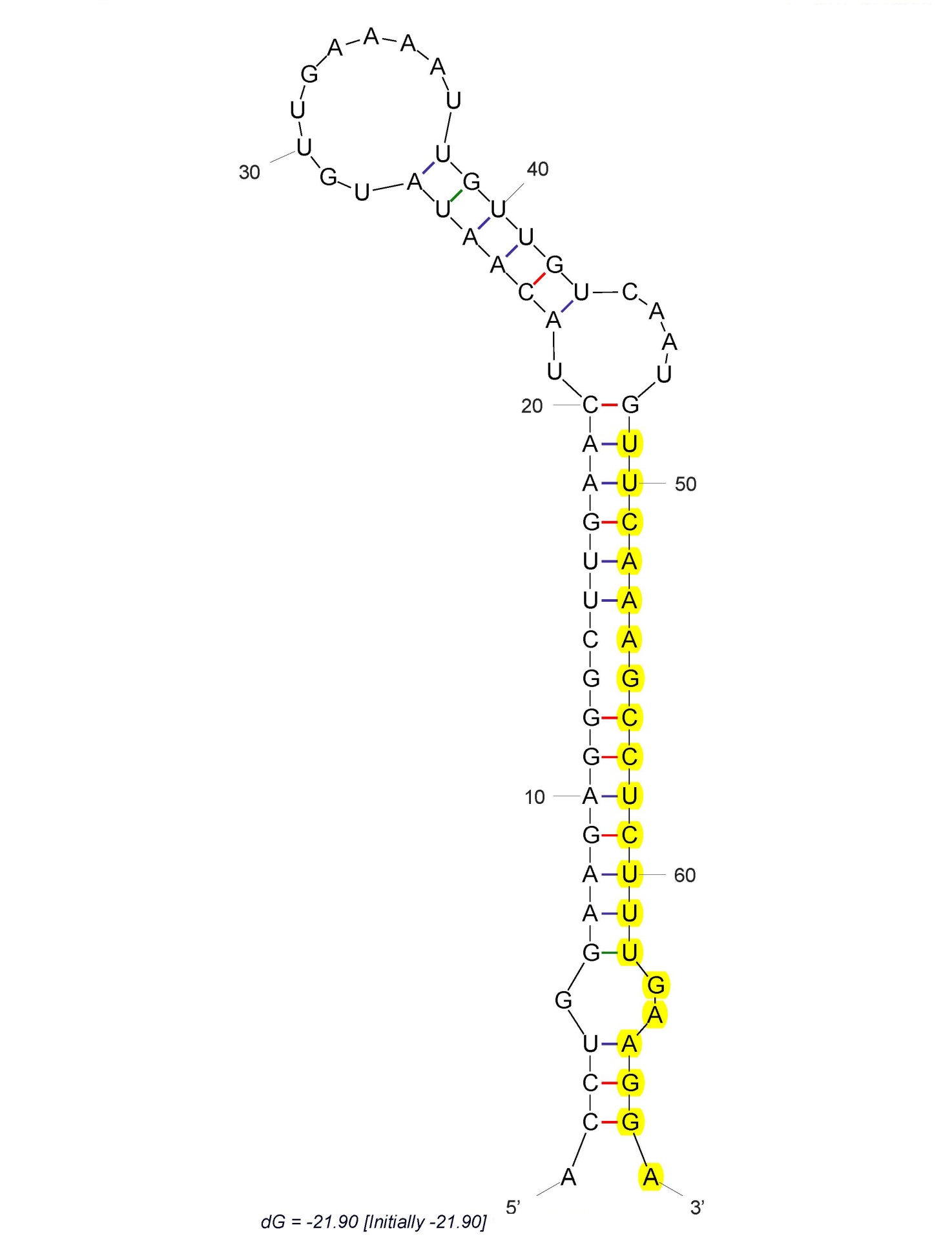


**Yellow sequences represent the mature miRNAs.**
