# Supplementary material for: Transcriptome analysis reveals ginsenosides biosynthetic genes, microRNAs and simple sequence repeats in Panax ginseng C. A. Meyer
Source: BMC Genomics. 2013 Apr 11;14:245. doi: 10.1186/1471-2164-14-245 (PMC3637502; doi:10.1186/1471-2164-14-245)
Supplement: Additional file 7 — miRNA potential target genes. DOCX document for the predicted miRNA target genes. [file 1471-2164-14-245-S7.docx]

**Table S4 - miRNA potential target genes**

| **miRNA family** | **miRNA** | **Unigene** | **Unigene annotation** |
| --- | --- | --- | --- |
| 1128 | pgi-miR1128 | root04345 | adh1-adh2 region |
| 1128 | pgi-miR1128 | root26558 | predicted protein |
| 1128 | pgi-miR1128 | stem32520 | hypothetical protein |
| 1128 | pgi-miR1128 | flower28672 | hypothetical protein |
| 1128 | pgi-miR1128 | flower17923 | Zinc transporter ZTP29 |
| 1439 | pgi-miR1439 | G9IMXQ301D0TKC | alpha-amylase |
| 1439 | pgi-miR1439 | G9IMXQ301CBT7A | MATE efflux family protein ALF5 |
| 5658 | pgi-miR5658 | G9IMXQ301AVMSH | DNA polymerase delta subunit |
| 5658 | pgi-miR5658 | G9IMXQ301BLGGT | GTP-binding protein |
| 5658 | pgi-miR5658 | G9IMXQ301CDX2L | 1-phosphatidylinositol-3-phosphate 5-kinase |
| 5658 | pgi-miR5658 | G9IMXQ301CWPAU | DNA binding / protein binding / transcription factor/ transcription regulator |
| 5658 | pgi-miR5658 | G9IMXQ301D4KW1 | ESCRT-II complex subunit VPS22 |
| 5658 | pgi-miR5658 | G9IMXQ301DMY6E | DNA polymerase delta subunit |
| 5658 | pgi-miR5658 | G9IMXQ301EJMD5 | ubiquitin carboxyl-terminal hydrolase |
| 5658 | pgi-miR5658 | G9IMXQ301ARFF8 | BEL1-like homeodomain protein |
| 5658 | pgi-miR5658 | G9IMXQ301C1F2U | Protein SEY1 |
| 5658 | pgi-miR5658 | G9IMXQ301CMST4 | Protein sey1 |
| 5658 | pgi-miR5658 | G9IMXQ301CWPAU | Protein WUSCHEL |
| 5658 | pgi-miR5658 | G9IMXQ301D2IDL | BTB/POZ domain-containing protein |
| 5658 | pgi-miR5658 | G9IMXQ301D4KW1 | Vacuolar protein sorting-associated protein |
| 5658 | pgi-miR5658 | G9IMXQ301D5Z33 | DEAD-box ATP-dependent RNA helicase |
| 5658 | pgi-miR5658 | G9IMXQ301DB45S | Tubby-like F-box protein 3 |
| 5658 | pgi-miR5658 | G9IMXQ301DDNLM | Peroxisomal 2,4-dienoyl-CoA reductase |
| 5658 | pgi-miR5658 | G9IMXQ301EJMD5 | Ubiquitin carboxyl-terminal hydrolase |
| 5658 | pgi-miR5658 | G9IMXQ302F4VOZ | ATP binding protein |
| 5658 | pgi-miR5658 | G9IMXQ302ITZVX | small nucleolar RNA-associated protein |
| 5658 | pgi-miR5658 | G9IMXQ302JLNJX | 1-phosphatidylinositol-3-phosphate 5-kinase |
| 5658 | pgi-miR5658 | G9IMXQ302H1D3T | Protein WUSCHEL |
| 5658 | pgi-miR5658 | G9IMXQ302HLSW8 | Cellulose synthase-like protein |
| 5658 | pgi-miR5658 | G9IMXQ302JLLCF | Zinc finger protein |
| 5658 | pgi-miR5658 | G8WERZI01COZRD | nucleotide binding protein |
| 5658 | pgi-miR5658 | G8WERZI01DW6V3 | phosphatidylinositol 4-kinase |
| 5658 | pgi-miR5658 | G8WERZI01DYHHZ | serine/threonine protein phosphatase |
| 5658 | pgi-miR5658 | G8WERZI01BQWWL | DEAD-box ATP-dependent RNA helicase |
| 5658 | pgi-miR5658 | G8WERZI01CXPF0 | Photosystem II 22 kDa protein, chloroplastic |
| 5658 | pgi-miR5658 | G8WERZI01DW6V3 | Phosphatidylinositol 4-kinase alpha |
| 5658 | pgi-miR5658 | G8WERZI02GBY9C | GC-rich sequence DNA-binding factor |
| 5658 | pgi-miR5658 | G8WERZI02FU9VM | DNA (cytosine-5)-methyltransferase |
| 5658 | pgi-miR5658 | G8WERZI02G8CV7 | RING-H2 finger protein |

**Table S4 - miRNA potential target genes** (*Continued*)

| **miRNA family** | **miRNA** | **Unigene** | **Unigene annotation** |
| --- | --- | --- | --- |
| 5658 | pgi-miR5658 | G8WERZI02IBACN | LRR receptor-like serine/threonine-protein kinase |
| 5658 | pgi-miR5658 | stem35181 | transcription factor, putative |
| 5658 | pgi-miR5658 | stem33718 | unnamed protein product |
| 5658 | pgi-miR5658 | stem31176 | late-embryogenesis abundant protein |
| 5658 | pgi-miR5658 | stem34992 | TSA: Arachis duranensis DurSNP_c13517.Ardu mRNA sequence |
| 403 | pgi-miR403b | stem26909 | eukaryotic translation initiation factor |
| 403 | pgi-miR403b | stem20177 | conserved hypothetical protein |
| 403 | pgi-miR403b | stem20177 | hypothetical protein |
| 172 | pgi-miR172 | G8WERZI02GKH8C | Floral homeotic protein APETALA2 |
| 5021 | pgi-miR5021 | G9IMXQ301CK3WU | Phospholipase |
| 5021 | pgi-miR5021 | G9IMXQ301CR1Z7 | Phytoene dehydrogenase |
| 5021 | pgi-miR5021 | G9IMXQ301CT7H5 | DNA polymerase delta catalytic subunit |
| 5021 | pgi-miR5021 | G9IMXQ301DAW18 | AP2-like factor, ANT lineage |
| 5021 | pgi-miR5021 | G9IMXQ301DBTQ7 | myb proto-oncogene protein |
| 5021 | pgi-miR5021 | G9IMXQ301DDNLM | 2,4-dienoyl-CoA reductase |
| 5021 | pgi-miR5021 | G9IMXQ301DVP5Q | pho1-like protein |
| 5021 | pgi-miR5021 | G9IMXQ301BHLXC | Probable polygalacturonase |
| 5021 | pgi-miR5021 | G9IMXQ301BR7I2 | L-ascorbate oxidase homolog |
| 5021 | pgi-miR5021 | G9IMXQ301CK3WU | Probable phospholipase A2 homolog |
| 5021 | pgi-miR5021 | G9IMXQ301CT7ND | Kinesin-related protein |
| 5021 | pgi-miR5021 | G9IMXQ301CUKVQ | Retrovirus-related Pol polyprotein from transposon |
| 5021 | pgi-miR5021 | G9IMXQ301EG73D | Probable polyamine oxidase |
| 5021 | pgi-miR5021 | G9IMXQ301EO106 | Probable receptor protein kinase |
| 5021 | pgi-miR5021 | G9IMXQ302F3QQ5 | K02540 minichromosome maintenance protein 2 |
| 5021 | pgi-miR5021 | G9IMXQ302G5OPV | kinesin heavy chain |
| 5021 | pgi-miR5021 | G9IMXQ302II89D | similar to pentatricopeptide (PPR) repeat-containing protein |
| 5021 | pgi-miR5021 | G9IMXQ302ITOO1 | zinc finger protein |
| 5021 | pgi-miR5021 | G9IMXQ302IWFNK | tryptophanyl-tRNA synthetase |
| 5021 | pgi-miR5021 | G9IMXQ302FLIK5 | dCTP pyrophosphatase |
| 5021 | pgi-miR5021 | G9IMXQ302G5OPV | Kinesin-related protein |
| 5021 | pgi-miR5021 | G9IMXQ302H7A88 | Tryptophan aminotransferase |
| 5021 | pgi-miR5021 | G9IMXQ302HY7VP | Tryptophanyl-tRNA synthetase |
| 5021 | pgi-miR5021 | G9IMXQ302ICS2M | Protein FAR1-RELATED SEQUENCE |
| 5021 | pgi-miR5021 | G9IMXQ302II89D | Pentatricopeptide repeat-containing protein |
| 5021 | pgi-miR5021 | G9IMXQ302IWFNK | Tryptophanyl-tRNA synthetase |
| 5021 | pgi-miR5021 | G9IMXQ302JWH32 | Probable receptor-like protein kinase |
| 5021 | pgi-miR5021 | G8WERZI01AL1ZM | 26S proteasome regulatory subunit N12 |
| 5021 | pgi-miR5021 | G8WERZI01ARBS7 | pre-mRNA-processing factor |

**Table S4 - miRNA potential target genes** (*Continued*)

| **miRNA family** | **miRNA** | **Unigene** | **Unigene annotation** |
| --- | --- | --- | --- |
| 5021 | pgi-miR5021 | G8WERZI01ASEWS | solute carrier family 30 (zinc transporter) |
| 5021 | pgi-miR5021 | G8WERZI01AZN2F | serine-threonine protein kinase |
| 5021 | pgi-miR5021 | G8WERZI01B1NP6 | DNA binding protein |
| 5021 | pgi-miR5021 | G8WERZI01B9GE3 | riboflavin biosynthesis protein-related |
| 5021 | pgi-miR5021 | G8WERZI01BRP5Q | amino acid binding protein |
| 5021 | pgi-miR5021 | G8WERZI01BYTFZ | small nucleolar RNA-associated protein |
| 5021 | pgi-miR5021 | G8WERZI01C2O5K | thiazole synthase |
| 5021 | pgi-miR5021 | G8WERZI01CF17X | splicing factor 45 |
| 5021 | pgi-miR5021 | G8WERZI01D0Q6P | valacyclovir hydrolase |
| 5021 | pgi-miR5021 | G8WERZI01EM9WN | inositol-tetrakisphosphate 1-kinase |
| 5021 | pgi-miR5021 | G8WERZI01EMSI7 | 60S ribosomal protein L24 |
| 5021 | pgi-miR5021 | G8WERZI01ESD7F | tuftelin-interacting protein 11 |
| 5021 | pgi-miR5021 | G8WERZI01AL1ZM | 26S proteasome non-ATPase regulatory subunit RPN12A |
| 5021 | pgi-miR5021 | G8WERZI01ALZBW | Thiazole biosynthetic enzyme |
| 5021 | pgi-miR5021 | G8WERZI01AZN2F | LRR receptor-like serine/threonine-protein kinase |
| 5021 | pgi-miR5021 | G8WERZI01CF17X | DNA-damage-repair/toleration protein |
| 5021 | pgi-miR5021 | G8WERZI01DA70L | Receptor-like protein |
| 5021 | pgi-miR5021 | G8WERZI01DCCTO | Lysosomal Pro-X carboxypeptidase |
| 5021 | pgi-miR5021 | G8WERZI01EJ1NH | TMV resistance protein |
| 5021 | pgi-miR5021 | G8WERZI01EUVS2 | Myb family transcription factor |
| 5021 | pgi-miR5021 | G8WERZI02FHXAE | Citrate-binding protein |
| 5021 | pgi-miR5021 | G8WERZI02HQV35 | Receptor-like protein |
| 3441.1 | pgi-miR3441.1 | G8WERZI02I7N7R | Os07g0673600 |
