# Supplementary material for: Transcriptome analysis reveals ginsenosides biosynthetic genes, microRNAs and simple sequence repeats in Panax ginseng C. A. Meyer
Source: BMC Genomics. 2013 Apr 11;14:245. doi: 10.1186/1471-2164-14-245 (PMC3637502; doi:10.1186/1471-2164-14-245)
Supplement: Additional file 8 — Occurrence of SSRs in P. ginseng unigenes. DOCX document for the summary of occurrence of SSRs in P. ginseng unigenes. [file 1471-2164-14-245-S8.docx]

**Table S5 - Occurrence of SSRs in *P. ginseng* unigenes**

| Repeat Motif | Repeat Number | | | | | | | | Total | % |
| --- | --- | --- | --- | --- | --- | --- | --- | --- | --- | --- |
|  | 4 | 5 | 6 | 7 | 8 | 9 | 10 | >10 |  |  |
| AC/GT | - | - | 241 | 165 | 98 | 76 | 40 | 186 | 806 | 6.2 |
| AG/CT | - | - | 1175 | 806 | 448 | 269 | 159 | 344 | 3201 | 24.5 |
| AT/AT | - | - | 743 | 554 | 452 | 286 | 213 | 386 | 2634 | 20.2 |
| CG/CG | - | - | 10 | 5 | 1 |  | 2 | 0 | 18 | 0.1 |
| AAC/GTT | - | 80 | 32 | 13 | 7 | 6 | 1 | 4 | 143 | 1.1 |
| AAG/CTT | - | 537 | 198 | 79 | 48 | 19 | 14 | 44 | 939 | 7.2 |
| AAT/ATT | - | 289 | 151 | 91 | 44 | 30 | 21 | 75 | 701 | 5.4 |
| ACC/GGT | - | 182 | 66 | 24 | 14 | 5 | 5 | 6 | 302 | 2.3 |
| ACG/CGT | - | 27 | 11 | 3 |  | 2 |  | 0 | 43 | 0.3 |
| ACT/AGT | - | 86 | 35 | 16 | 15 | 3 | 6 | 12 | 173 | 1.3 |
| AGC/CTG | - | 209 | 94 | 58 | 26 | 20 | 7 | 6 | 420 | 3.2 |
| AGG/CCT | - | 168 | 99 | 53 | 14 | 11 | 5 | 1 | 351 | 2.7 |
| ATC/ATG | - | 229 | 85 | 64 | 58 | 20 | 8 | 14 | 478 | 3.7 |
| CCG/CGG | - | 61 | 15 | 7 |  | 2 |  | 0 | 85 | 0.7 |
| AAAC/GTTT | 70 | 16 | 5 | 2 |  |  |  | 0 | 93 | 0.7 |
| AAAG/CTTT | 78 | 29 | 22 | 8 | 4 |  |  | 0 | 141 | 1.1 |
| AAAT/ATTT | 230 | 56 | 15 | 3 | 2 |  | 1 | 1 | 308 | 2.4 |
| AACC/GGTT | 21 | 6 |  |  |  |  |  | 0 | 27 | 0.2 |
| AACG/CGTT | 6 | 3 |  |  |  |  |  | 0 | 9 | 0.1 |
| AACT/AGTT | 13 | 1 | 5 |  |  |  |  | 0 | 19 | 0.1 |
| AAGC/CTTG | 24 | 1 |  | 1 |  |  |  | 0 | 26 | 0.2 |
| AAGG/CCTT | 26 | 3 | 4 | 5 | 1 |  |  | 1 | 40 | 0.3 |
| AAGT/ACTT | 1 |  |  |  |  |  |  | 0 | 1 | 0.0 |
| AATC/ATTG | 24 | 3 |  |  |  |  |  | 0 | 27 | 0.2 |
| AATG/ATTC | 20 | 2 | 1 |  |  |  |  | 0 | 23 | 0.2 |
| AATT/AATT | 44 | 4 |  |  |  |  |  | 0 | 48 | 0.4 |
| ACAG/CTGT | 15 | 4 | 2 |  | 1 |  |  | 0 | 22 | 0.2 |
| ACAT/ATGT | 61 | 23 | 14 | 16 |  |  |  | 2 | 116 | 0.9 |
| ACCC/GGGT | 2 | 3 | 1 | 1 |  |  |  | 0 | 7 | 0.1 |
| ACCG/CGGT | 5 | 3 | 1 |  |  |  |  | 0 | 9 | 0.1 |
| ACCT/AGGT | 10 | 2 | 1 |  |  |  |  | 0 | 13 | 0.1 |
| ACGC/CGTG | 2 | 3 | 1 | 1 | 1 |  |  | 0 | 8 | 0.1 |
| ACGG/CCGT | 6 | 3 | 1 |  |  |  |  | 0 | 10 | 0.1 |
| ACGT/ACGT | 6 | 3 | 2 |  | 1 | 1 |  | 2 | 15 | 0.1 |
| ACTC/AGTG | 18 | 7 | 2 | 4 | 2 | 1 |  | 0 | 34 | 0.3 |
| ACTG/AGTC | 6 | 4 | 1 |  |  | 1 |  | 0 | 12 | 0.1 |
| AGAT/ATCT | 65 | 20 | 11 | 6 | 8 | 3 |  | 1 | 114 | 0.9 |
| AGCC/CTGG | 24 | 9 | 5 | 2 | 1 |  |  | 0 | 41 | 0.3 |
| AGCG/CGCT | 11 | 2 | 2 |  |  |  |  | 0 | 15 | 0.1 |
| AGCT/AGCT | 72 | 9 | 5 |  |  |  |  | 0 | 86 | 0.7 |
| Repeat Motif | Repeat Number | | | | | | | | Total | % |
|  | 4 | 5 | 6 | 7 | 8 | 9 | 10 | >10 |  |  |
| AGGC/CCTG | 24 | 6 | 5 | 5 |  |  |  | 0 | 40 | 0.3 |
| AGGG/CCCT | 42 | 15 | 8 | 3 |  |  |  | 0 | 68 | 0.5 |
| ATCC/ATGG | 21 | 6 | 2 |  | 1 | 1 |  | 0 | 31 | 0.2 |
| ATCG/ATCG | 6 | 1 |  |  |  |  |  | 0 | 7 | 0.1 |
| ATGC/ATGC | 27 | 5 |  |  |  |  |  | 0 | 32 | 0.2 |
| CCCG/CGGG | 4 | 2 |  |  |  |  |  | 0 | 6 | 0.0 |
| CCGG/CCGG | 6 | 1 |  |  |  |  |  | 0 | 7 | 0.1 |
| AAAAC/GTTTT | 13 | 6 |  | 1 |  |  |  | 0 | 20 | 0.2 |
| AAAAG/CTTTT | 12 | 2 | 3 |  |  |  |  | 0 | 17 | 0.1 |
| AAAAT/ATTTT | 34 | 3 | 1 | 1 |  |  | 1 | 0 | 40 | 0.3 |
| AAACC/GGTTT | 29 | 5 |  |  |  |  |  | 0 | 34 | 0.3 |
| AAACT/AGTTT | 1 |  |  |  |  |  |  | 0 | 1 | 0.0 |
| AAAGC/CTTTG | 5 |  | 1 |  |  |  |  | 0 | 6 | 0.0 |
| AAAGG/CCTTT | 8 | 5 | 3 |  |  |  |  | 0 | 16 | 0.1 |
| AAAGT/ACTTT |  | 1 |  |  |  |  |  | 0 | 1 | 0.0 |
| AAATC/ATTTG | 8 | 1 |  |  |  |  |  | 0 | 9 | 0.1 |
| AAATG/ATTTC |  | 1 |  |  |  |  |  | 0 | 1 | 0.0 |
| AAATT/AATTT | 4 | 1 |  |  |  |  |  | 0 | 5 | 0.0 |
| AACAC/GTGTT | 2 | 2 |  |  |  |  |  | 0 | 4 | 0.0 |
| AACAG/CTGTT | 9 | 2 |  |  |  |  |  | 0 | 11 | 0.1 |
| AACCC/GGGTT | 6 | 1 |  |  |  |  |  | 0 | 7 | 0.1 |
| AACCG/CGGTT | 1 |  |  |  |  |  |  | 0 | 1 | 0.0 |
| AACCT/AGGTT |  | 1 | 1 |  |  |  |  | 0 | 2 | 0.0 |
| AACGG/CCGTT | 1 |  |  |  |  |  |  | 0 | 1 | 0.0 |
| AACTC/AGTTG | 4 |  |  |  |  |  |  | 0 | 4 | 0.0 |
| AACTG/AGTTC | 2 | 2 |  |  |  |  |  | 0 | 4 | 0.0 |
| AACTT/AAGTT | 1 |  |  |  |  |  |  | 0 | 1 | 0.0 |
| AAGAC/CTTGT | 1 |  |  |  |  |  |  | 0 | 1 | 0.0 |
| AAGAG/CTCTT | 14 | 7 | 6 |  |  |  |  | 0 | 27 | 0.2 |
| AAGCC/CTTGG | 6 | 1 |  |  |  |  |  | 0 | 7 | 0.1 |
| AAGCT/AGCTT | 4 |  |  |  |  |  |  | 0 | 4 | 0.0 |
| AAGGC/CCTTG |  |  |  | 1 |  |  |  | 0 | 1 | 0.0 |
| AAGGG/CCCTT | 7 | 1 | 1 | 1 |  |  |  | 0 | 10 | 0.1 |
| AAGTG/ACTTC | 2 |  |  |  |  |  |  | 0 | 2 | 0.0 |
| AATAC/ATTGT | 2 | 1 | 1 |  |  |  |  | 0 | 4 | 0.0 |
| AATAG/ATTCT | 4 | 1 |  |  |  |  |  | 0 | 5 | 0.0 |
| AATAT/ATATT | 4 |  | 1 |  |  | 1 |  | 0 | 6 | 0.0 |
| AATCC/ATTGG | 1 | 6 | 2 | 1 |  |  |  | 0 | 10 | 0.1 |
| AATCT/AGATT | 1 |  |  | 1 |  |  |  | 0 | 2 | 0.0 |
| AATGC/ATTGC | 1 |  |  |  |  |  |  | 0 | 1 | 0.0 |
| AATGG/ATTCC | 5 | 1 |  |  |  |  |  | 0 | 6 | 0.0 |
| AATGT/ACATT | 2 | 1 |  |  |  |  |  | 0 | 3 | 0.0 |
| Repeat Motif | Repeat Number | | | | | | | | Total | % |
|  | 4 | 5 | 6 | 7 | 8 | 9 | 10 | >10 |  |  |
| AATTC/AATTG | 6 | 3 |  |  |  |  |  | 0 | 9 | 0.1 |
| ACACC/GGTGT | 4 |  |  |  |  |  |  | 0 | 4 | 0.0 |
| ACACG/CGTGT | 1 |  |  |  |  |  |  | 0 | 1 | 0.0 |
| ACACT/AGTGT | 3 |  |  |  |  |  |  | 0 | 3 | 0.0 |
| ACAGC/CTGTG | 4 | 2 | 1 |  |  |  |  | 0 | 7 | 0.1 |
| ACAGG/CCTGT | 5 |  |  | 2 |  |  |  | 0 | 7 | 0.1 |
| ACAGT/ACTGT | 1 | 1 |  |  |  |  |  | 0 | 2 | 0.0 |
| ACATC/ATGTG | 4 |  | 1 |  |  |  |  | 0 | 5 | 0.0 |
| ACATG/ATGTC | 2 |  |  |  |  |  |  | 0 | 2 | 0.0 |
| ACCAG/CTGGT | 5 | 2 | 2 |  |  |  |  | 0 | 9 | 0.1 |
| ACCAT/ATGGT | 2 |  | 1 |  |  |  |  | 0 | 3 | 0.0 |
| ACCCC/GGGGT | 8 | 5 | 2 | 1 | 1 |  |  | 0 | 17 | 0.1 |
| ACCCG/CGGGT | 3 |  |  |  |  |  |  | 0 | 3 | 0.0 |
| ACCCT/AGGGT | 4 | 1 | 1 | 1 |  |  |  | 0 | 7 | 0.1 |
| ACCGT/ACGGT | 1 |  |  |  |  |  |  | 0 | 1 | 0.0 |
| ACCTC/AGGTG | 5 | 2 |  |  |  |  |  | 0 | 7 | 0.1 |
| ACCTG/AGGTC | 2 | 1 |  |  |  |  |  | 0 | 3 | 0.0 |
| ACGAG/CGTCT | 4 |  |  |  |  |  |  | 0 | 4 | 0.0 |
| ACGAT/ATCGT |  | 1 |  |  |  |  |  | 0 | 1 | 0.0 |
| ACGCC/CGTGG | 1 |  |  |  |  |  |  | 0 | 1 | 0.0 |
| ACGGC/CCGTG | 1 | 1 |  |  |  |  |  | 0 | 2 | 0.0 |
| ACGGG/CCCGT | 1 |  |  | 1 |  |  |  | 0 | 2 | 0.0 |
| ACTAG/AGTCT | 1 | 3 |  |  |  |  |  | 0 | 4 | 0.0 |
| ACTAT/AGTAT | 3 |  |  |  |  |  |  | 0 | 3 | 0.0 |
| ACTCC/AGTGG | 4 | 1 |  | 1 |  |  |  | 0 | 6 | 0.0 |
| ACTCG/AGTCG | 3 |  |  |  |  |  |  | 0 | 3 | 0.0 |
| ACTCT/AGAGT | 3 | 3 |  |  |  |  |  | 0 | 6 | 0.0 |
| ACTGC/AGTGC | 1 |  |  |  |  |  |  | 0 | 1 | 0.0 |
| ACTGG/AGTCC | 4 | 1 |  |  |  |  |  | 0 | 5 | 0.0 |
| AGAGC/CTCTG | 10 | 3 |  |  |  |  |  | 0 | 13 | 0.1 |
| AGAGG/CCTCT | 14 | 4 | 3 | 2 |  |  |  | 0 | 23 | 0.2 |
| AGATC/ATCTG | 2 | 2 |  |  |  |  |  | 0 | 4 | 0.0 |
| AGATG/ATCTC | 2 | 2 |  |  |  |  |  | 0 | 4 | 0.0 |
| AGCAT/ATGCT | 5 | 3 |  |  |  |  |  | 0 | 8 | 0.1 |
| AGCCC/CTGGG | 3 | 2 | 4 | 1 |  |  |  | 0 | 10 | 0.1 |
| AGCCT/AGGCT | 2 | 1 |  |  |  |  |  | 0 | 3 | 0.0 |
| AGCGC/CGCTG | 1 |  |  |  |  |  |  | 0 | 1 | 0.0 |
| AGCTC/AGCTG | 2 | 1 | 1 |  |  |  |  | 0 | 4 | 0.0 |
| AGGAT/ATCCT | 4 | 1 |  |  |  |  |  | 0 | 5 | 0.0 |
| AGGCC/CCTGG | 4 |  |  |  |  |  |  | 0 | 4 | 0.0 |
| AGGCG/CCTCG | 6 | 1 | 1 |  |  |  |  | 0 | 8 | 0.1 |
| AGGGC/CCCTG | 6 | 4 |  |  |  |  |  | 0 | 10 | 0.1 |
| Repeat Motif | Repeat Number | | | | | | | | Total | % |
|  | 4 | 5 | 6 | 7 | 8 | 9 | 10 | >10 |  |  |
| AGGGG/CCCCT | 3 | 6 | 1 |  |  |  |  | 0 | 10 | 0.1 |
| ATATC/ATATG | 1 | 2 |  |  |  |  |  | 0 | 3 | 0.0 |
| ATCCC/ATGGG | 9 | 1 | 1 |  |  |  |  | 0 | 11 | 0.1 |
| ATCCG/ATCGG | 2 | 1 |  |  |  |  |  | 0 | 3 | 0.0 |
| ATGCC/ATGGC | 2 | 2 | 1 |  |  |  |  | 0 | 5 | 0.0 |
| CCCGG/CCGGG |  | 1 |  |  |  |  |  | 0 | 1 | 0.0 |
| AAAAAC/GTTTTT | 9 | 1 |  |  |  |  |  | 0 | 10 | 0.1 |
| AAAAAG/CTTTTT | 5 | 1 |  |  |  |  |  | 0 | 6 | 0.0 |
| AAAAAT/ATTTTT | 13 | 5 |  |  |  |  | 1 | 0 | 19 | 0.1 |
| AAAACC/GGTTTT | 8 | 2 |  |  |  |  |  | 0 | 10 | 0.1 |
| AAAACT/AGTTTT | 1 | 1 |  |  |  |  |  | 0 | 2 | 0.0 |
| AAAAGC/CTTTTG | 3 |  |  |  |  |  |  | 0 | 3 | 0.0 |
| AAAAGG/CCTTTT | 4 | 5 |  |  |  |  |  | 0 | 9 | 0.1 |
| AAAAGT/ACTTTT | 1 |  |  |  |  |  |  | 0 | 1 | 0.0 |
| AAAATC/ATTTTG | 1 | 1 | 1 |  |  |  |  | 0 | 3 | 0.0 |
| AAAATG/ATTTTC | 3 | 1 |  |  |  |  |  | 0 | 4 | 0.0 |
| AAAATT/AATTTT | 1 | 1 |  |  |  |  |  | 0 | 2 | 0.0 |
| AAACAC/GTGTTT | 2 |  |  |  | 1 |  |  | 0 | 3 | 0.0 |
| AAACAG/CTGTTT |  |  | 1 |  |  |  |  | 0 | 1 | 0.0 |
| AAACCC/GGGTTT | 5 | 3 |  | 1 |  |  |  | 0 | 9 | 0.1 |
| AAACCG/CGGTTT | 1 |  |  |  |  |  |  | 0 | 1 | 0.0 |
| AAAGAG/CTCTTT | 11 | 4 | 1 |  |  |  |  | 0 | 16 | 0.1 |
| AAAGCC/CTTTGG | 4 | 1 | 1 |  |  |  |  | 0 | 6 | 0.0 |
| AAAGGG/CCCTTT | 4 | 3 |  |  |  |  |  | 0 | 7 | 0.1 |
| AAAGGT/ACCTTT | 1 | 1 |  |  |  |  |  | 0 | 2 | 0.0 |
| AAAGTC/ACTTTG |  |  | 1 |  |  |  |  | 0 | 1 | 0.0 |
| AAAGTG/ACTTTC |  | 1 |  |  |  |  |  | 0 | 1 | 0.0 |
| AAATAG/ATTTCT | 2 | 1 |  |  |  |  |  | 0 | 3 | 0.0 |
| AAATAT/ATATTT |  |  | 1 |  |  |  |  | 0 | 1 | 0.0 |
| AAATCC/ATTTGG | 4 | 2 |  |  |  |  |  | 0 | 6 | 0.0 |
| AAATCG/ATTTCG | 1 |  |  |  |  |  |  | 0 | 1 | 0.0 |
| AAATGC/ATTTGC | 2 | 2 |  |  |  |  |  | 0 | 4 | 0.0 |
| AAATGG/ATTTCC | 6 | 2 |  |  |  |  |  | 0 | 8 | 0.1 |
| AAATGT/ACATTT | 2 |  |  |  |  |  |  | 0 | 2 | 0.0 |
| AAATTC/AATTTG | 2 |  |  |  |  |  |  | 0 | 2 | 0.0 |
| AAATTG/AATTTC | 1 |  |  |  |  |  |  | 0 | 1 | 0.0 |
| AAATTT/AAATTT | 1 |  |  |  |  |  |  | 0 | 1 | 0.0 |
| AACAAG/CTTGTT | 1 |  |  |  |  |  |  | 0 | 1 | 0.0 |
| AACACC/GGTGTT | 1 | 1 |  |  |  |  |  | 0 | 2 | 0.0 |
| AACACT/AGTGTT | 1 |  |  |  |  |  |  | 0 | 1 | 0.0 |
| AACAGC/CTGTTG | 10 | 1 |  | 1 |  | 1 | 1 | 0 | 14 | 0.1 |
| AACATC/ATGTTG | 1 |  |  |  |  |  |  | 0 | 1 | 0.0 |
| Repeat Motif | Repeat Number | | | | | | | | Total | % |
|  | 4 | 5 | 6 | 7 | 8 | 9 | 10 | >10 |  |  |
| AACCAC/GGTTGT | 1 |  |  |  |  |  |  | 0 | 1 | 0.0 |
| AACCAG/CTGGTT |  | 1 |  |  |  |  |  | 0 | 1 | 0.0 |
| AACCAT/ATGGTT | 1 | 1 |  | 1 |  | 1 |  | 1 | 5 | 0.0 |
| AACCCG/CGGGTT | 2 |  |  |  |  |  |  | 0 | 2 | 0.0 |
| AACCCT/AGGGTT | 4 | 1 |  | 1 |  |  |  | 0 | 6 | 0.0 |
| AACCGC/CGGTTG | 1 |  |  |  |  |  |  | 0 | 1 | 0.0 |
| AACCGG/CCGGTT | 1 |  |  |  |  |  |  | 0 | 1 | 0.0 |
| AACCTC/AGGTTG | 1 |  |  |  |  |  |  | 0 | 1 | 0.0 |
| AACTAC/AGTTGT | 3 | 1 |  |  |  |  |  | 0 | 4 | 0.0 |
| AACTAG/AGTTCT | 1 |  |  |  |  |  |  | 0 | 1 | 0.0 |
| AACTCC/AGTTGG | 2 |  |  |  |  |  |  | 0 | 2 | 0.0 |
| AACTCG/AGTTCG | 1 |  |  |  |  |  |  | 0 | 1 | 0.0 |
| AACTGC/AGTTGC |  |  |  | 1 |  |  |  | 0 | 1 | 0.0 |
| AACTGG/AGTTCC | 1 |  |  |  |  |  |  | 0 | 1 | 0.0 |
| AACTTC/AAGTTG |  | 1 |  |  |  |  |  | 0 | 1 | 0.0 |
| AAGACC/CTTGGT | 1 |  |  |  |  |  |  | 0 | 1 | 0.0 |
| AAGACG/CGTCTT |  |  | 1 |  |  |  |  | 0 | 1 | 0.0 |
| AAGAGC/CTCTTG | 2 |  |  |  |  |  |  | 0 | 2 | 0.0 |
| AAGAGG/CCTCTT | 10 | 6 | 3 |  |  |  |  | 0 | 19 | 0.1 |
| AAGATC/ATCTTG | 3 |  |  |  |  |  |  | 0 | 3 | 0.0 |
| AAGATG/ATCTTC | 12 | 7 | 1 |  |  |  |  | 0 | 20 | 0.2 |
| AAGCAC/CTTGTG |  | 1 |  |  |  |  |  | 0 | 1 | 0.0 |
| AAGCAG/CTGCTT |  | 4 | 2 |  | 1 |  |  | 0 | 7 | 0.1 |
| AAGCCC/CTTGGG |  |  |  | 1 |  |  |  | 0 | 1 | 0.0 |
| AAGCGG/CCGCTT | 1 |  |  |  |  |  |  | 0 | 1 | 0.0 |
| AAGCTC/AGCTTG | 4 |  |  |  |  |  |  | 0 | 4 | 0.0 |
| AAGGAC/CCTTGT | 1 | 1 |  |  |  |  |  | 0 | 2 | 0.0 |
| AAGGAG/CCTTCT | 7 | 3 | 2 |  | 2 |  |  | 0 | 14 | 0.1 |
| AAGGAT/ATCCTT |  |  |  | 1 |  |  |  | 0 | 1 | 0.0 |
| AAGGGC/CCCTTG | 2 |  |  |  |  |  |  | 0 | 2 | 0.0 |
| AAGGGG/CCCCTT | 3 |  |  |  |  |  |  | 0 | 3 | 0.0 |
| AAGGTG/ACCTTC | 2 |  | 1 |  |  |  |  | 0 | 3 | 0.0 |
| AAGTAG/ACTTCT | 5 |  |  |  |  |  |  | 0 | 5 | 0.0 |
| AAGTCC/ACTTGG | 1 |  |  |  |  |  |  | 0 | 1 | 0.0 |
| AAGTGC/ACTTGC | 1 |  |  |  |  |  |  | 0 | 1 | 0.0 |
| AAGTGG/ACTTCC | 6 |  |  |  |  |  |  | 0 | 6 | 0.0 |
| AATACT/AGTATT | 4 |  |  | 2 |  |  |  | 0 | 6 | 0.0 |
| AATAGC/ATTGCT | 1 | 1 |  |  |  |  |  | 0 | 2 | 0.0 |
| AATAGG/ATTCCT | 2 |  | 1 |  |  |  |  | 0 | 3 | 0.0 |
| AATAGT/ACTATT | 5 | 1 |  | 1 |  |  |  | 0 | 7 | 0.1 |
| AATATG/ATATTC | 1 |  |  |  |  |  |  | 0 | 1 | 0.0 |
| AATATT/AATATT |  |  |  |  | 1 |  |  | 1 | 2 | 0.0 |
| Repeat Motif | Repeat Number | | | | | | | | Total | % |
|  | 4 | 5 | 6 | 7 | 8 | 9 | 10 | >10 |  |  |
| AATCAT/ATGATT | 1 | 5 | 2 | 4 |  |  |  | 0 | 12 | 0.1 |
| AATCCC/ATTGGG | 2 | 1 |  |  |  |  |  | 0 | 3 | 0.0 |
| AATCCG/ATTCGG | 2 |  |  |  |  |  |  | 0 | 2 | 0.0 |
| AATCGG/ATTCCG | 2 | 4 |  |  | 1 |  |  | 0 | 7 | 0.1 |
| AATCTC/AGATTG |  |  | 1 |  |  |  |  | 0 | 1 | 0.0 |
| AATCTG/AGATTC | 1 | 1 |  |  |  |  |  | 0 | 2 | 0.0 |
| AATGAT/ATCATT | 1 |  |  |  |  |  |  | 0 | 1 | 0.0 |
| AATGCC/ATTGGC | 1 | 1 |  |  |  |  |  | 0 | 2 | 0.0 |
| AATGGC/ATTGCC | 4 | 1 |  | 1 |  |  |  | 0 | 6 | 0.0 |
| AATGGG/ATTCCC | 4 | 4 | 3 |  |  |  |  | 0 | 11 | 0.1 |
| AATGGT/ACCATT |  |  |  | 1 | 2 |  |  | 0 | 3 | 0.0 |
| AATGTG/ACATTC |  |  |  |  |  |  |  | 1 | 1 | 0.0 |
| AATTAC/AATTGT | 1 |  |  |  |  |  |  | 0 | 1 | 0.0 |
| AATTAG/AATTCT | 1 | 1 |  |  |  |  |  | 0 | 2 | 0.0 |
| AATTCC/AATTGG | 6 | 2 | 1 |  |  |  |  | 0 | 9 | 0.1 |
| AATTGC/AATTGC | 1 |  |  |  |  |  |  | 0 | 1 | 0.0 |
| ACACAG/CTGTGT |  | 1 |  |  |  |  | 2 | 0 | 3 | 0.0 |
| ACACCC/GGGTGT | 1 |  | 1 | 1 | 1 |  |  | 0 | 4 | 0.0 |
| ACACCG/CGGTGT | 1 |  |  |  |  |  |  | 0 | 1 | 0.0 |
| ACACCT/AGGTGT | 1 |  |  |  |  |  |  | 0 | 1 | 0.0 |
| ACACTC/AGTGTG | 1 |  |  |  |  | 1 |  | 0 | 2 | 0.0 |
| ACAGAT/ATCTGT |  | 1 |  |  |  |  |  | 0 | 1 | 0.0 |
| ACAGCC/CTGTGG | 2 | 3 |  |  |  |  |  | 0 | 5 | 0.0 |
| ACAGCT/AGCTGT | 1 | 1 |  |  |  |  |  | 0 | 2 | 0.0 |
| ACAGGC/CCTGTG | 3 |  |  |  |  |  |  | 0 | 3 | 0.0 |
| ACAGGG/CCCTGT | 3 |  |  |  |  |  |  | 0 | 3 | 0.0 |
| ACAGTG/ACTGTC |  |  |  |  |  | 1 |  | 0 | 1 | 0.0 |
| ACATAT/ATATGT | 5 |  | 4 |  |  |  |  | 0 | 9 | 0.1 |
| ACATCC/ATGTGG | 1 |  |  |  |  |  |  | 0 | 1 | 0.0 |
| ACATCT/AGATGT | 2 |  | 1 |  |  |  |  | 0 | 3 | 0.0 |
| ACATGC/ATGTGC | 1 | 2 | 2 |  |  |  |  | 0 | 5 | 0.0 |
| ACATGG/ATGTCC | 2 |  |  |  |  |  |  | 0 | 2 | 0.0 |
| ACCACG/CGTGGT | 1 |  | 1 |  |  |  |  | 0 | 2 | 0.0 |
| ACCACT/AGTGGT | 1 | 1 |  | 1 |  |  |  | 0 | 3 | 0.0 |
| ACCAGC/CTGGTG | 6 | 1 | 2 |  |  |  |  | 0 | 9 | 0.1 |
| ACCAGG/CCTGGT |  | 1 |  |  |  |  |  | 0 | 1 | 0.0 |
| ACCAGT/ACTGGT | 2 | 1 |  |  |  |  |  | 0 | 3 | 0.0 |
| ACCATC/ATGGTG | 3 | 5 | 3 | 1 |  |  |  | 0 | 12 | 0.1 |
| ACCATG/ATGGTC | 1 |  |  |  |  |  |  | 0 | 1 | 0.0 |
| ACCCAG/CTGGGT | 1 | 4 |  |  |  |  |  | 0 | 5 | 0.0 |
| ACCCAT/ATGGGT | 1 | 1 |  |  |  |  |  | 0 | 2 | 0.0 |
| ACCCCC/GGGGGT | 3 |  |  |  |  |  |  | 0 | 3 | 0.0 |
| Repeat Motif | Repeat Number | | | | | | | | Total | % |
|  | 4 | 5 | 6 | 7 | 8 | 9 | 10 | >10 |  |  |
| ACCCCG/CGGGGT |  | 1 |  |  |  |  |  | 0 | 1 | 0.0 |
| ACCCCT/AGGGGT | 4 | 1 |  |  |  |  |  | 0 | 5 | 0.0 |
| ACCCTC/AGGGTG | 1 | 2 | 1 |  |  |  |  | 0 | 4 | 0.0 |
| ACCCTG/AGGGTC |  | 1 | 1 | 1 |  |  |  | 0 | 3 | 0.0 |
| ACCGAT/ATCGGT | 1 |  |  |  |  |  |  | 0 | 1 | 0.0 |
| ACCGCC/CGGTGG | 2 | 2 |  |  |  |  |  | 0 | 4 | 0.0 |
| ACCGGC/CCGGTG |  |  | 1 |  |  |  |  | 0 | 1 | 0.0 |
| ACCGGG/CCCGGT | 1 |  |  |  |  |  |  | 0 | 1 | 0.0 |
| ACCGGT/ACCGGT | 1 |  |  |  |  |  |  | 0 | 1 | 0.0 |
| ACCGTC/ACGGTG | 1 |  | 1 |  |  |  |  | 0 | 2 | 0.0 |
| ACCGTG/ACGGTC | 1 |  |  | 1 |  |  |  | 0 | 2 | 0.0 |
| ACCTAG/AGGTCT | 1 | 1 |  |  |  |  |  | 0 | 2 | 0.0 |
| ACCTAT/AGGTAT |  | 1 |  |  |  |  |  | 0 | 1 | 0.0 |
| ACCTCC/AGGTGG | 11 |  | 3 | 1 |  |  |  | 0 | 15 | 0.1 |
| ACCTCG/AGGTCG |  | 1 | 1 |  |  |  |  | 0 | 2 | 0.0 |
| ACCTGC/AGGTGC | 1 |  |  |  |  |  |  | 0 | 1 | 0.0 |
| ACCTGG/AGGTCC | 1 | 2 | 1 | 1 |  |  |  | 0 | 5 | 0.0 |
| ACGAGG/CCTCGT | 1 | 2 | 1 |  |  |  |  | 0 | 4 | 0.0 |
| ACGATG/ATCGTC | 1 | 6 |  | 1 |  |  |  | 1 | 9 | 0.1 |
| ACGCAG/CGTCTG | 2 |  |  |  |  |  |  | 0 | 2 | 0.0 |
| ACGCAT/ATGCGT |  |  |  |  |  | 1 |  | 1 | 2 | 0.0 |
| ACGCCC/CGTGGG |  | 2 |  |  |  |  |  | 0 | 2 | 0.0 |
| ACGCCG/CGGCGT | 1 |  |  |  |  |  |  | 0 | 1 | 0.0 |
| ACGGAG/CCGTCT | 1 | 1 |  |  |  |  |  | 0 | 2 | 0.0 |
| ACGTCC/ACGTGG |  | 1 |  |  |  |  |  | 0 | 1 | 0.0 |
| ACTAGG/AGTCCT |  |  |  | 1 |  |  |  | 0 | 1 | 0.0 |
| ACTAGT/ACTAGT | 3 | 1 | 1 |  |  |  |  | 0 | 5 | 0.0 |
| ACTATG/AGTCAT |  |  | 1 |  |  |  |  | 0 | 1 | 0.0 |
| ACTCCC/AGTGGG | 1 | 2 |  |  |  |  |  | 0 | 3 | 0.0 |
| ACTCCG/AGTCGG | 1 |  |  | 1 |  |  |  | 0 | 2 | 0.0 |
| ACTCCT/AGGAGT | 2 | 1 |  |  |  |  |  | 0 | 3 | 0.0 |
| ACTCGC/AGTGCG | 1 |  |  |  |  |  |  | 0 | 1 | 0.0 |
| ACTCTC/AGAGTG | 1 | 3 |  |  |  |  |  | 0 | 4 | 0.0 |
| ACTGAG/AGTCTC | 2 |  |  |  |  |  |  | 0 | 2 | 0.0 |
| ACTGAT/AGTATC | 1 |  |  |  |  |  |  | 0 | 1 | 0.0 |
| ACTGCC/AGTGGC | 3 | 1 |  | 1 |  |  |  | 0 | 5 | 0.0 |
| ACTGCT/AGCAGT | 1 | 4 | 1 |  |  |  |  | 0 | 6 | 0.0 |
| ACTGGC/AGTGCC | 1 |  |  |  |  |  |  | 0 | 1 | 0.0 |
| ACTGGG/AGTCCC | 1 |  | 1 |  |  |  |  | 0 | 2 | 0.0 |
| AGAGAT/ATCTCT | 2 | 1 | 1 |  |  |  |  | 0 | 4 | 0.0 |
| AGAGCC/CTCTGG | 2 |  | 1 |  |  |  |  | 0 | 3 | 0.0 |
| AGAGCG/CGCTCT | 2 |  |  |  |  |  |  | 0 | 2 | 0.0 |
| Repeat Motif | Repeat Number | | | | | | | | Total | % |
|  | 4 | 5 | 6 | 7 | 8 | 9 | 10 | >10 |  |  |
| AGAGCT/AGCTCT | 1 |  |  |  |  |  |  | 0 | 1 | 0.0 |
| AGAGGC/CCTCTG | 1 |  |  |  |  |  |  | 0 | 1 | 0.0 |
| AGAGGG/CCCTCT | 22 | 11 | 2 |  |  |  |  | 0 | 35 | 0.3 |
| AGATAT/ATATCT | 3 | 2 |  | 1 |  |  |  | 0 | 6 | 0.0 |
| AGATCC/ATCTGG | 1 | 1 |  |  |  |  |  | 0 | 2 | 0.0 |
| AGATCG/ATCTCG | 2 | 4 |  |  |  |  |  | 0 | 6 | 0.0 |
| AGATCT/AGATCT |  | 1 |  |  |  |  |  | 0 | 1 | 0.0 |
| AGATGC/ATCTGC |  | 1 |  |  |  |  |  | 0 | 1 | 0.0 |
| AGATGG/ATCTCC | 9 | 3 | 1 | 1 | 2 |  |  | 0 | 16 | 0.1 |
| AGCAGG/CCTGCT | 7 | 4 | 1 |  |  |  |  | 0 | 12 | 0.1 |
| AGCATC/ATGCTG | 1 | 2 | 1 |  |  |  |  | 0 | 4 | 0.0 |
| AGCATG/ATGCTC | 2 |  |  |  |  |  |  | 0 | 2 | 0.0 |
| AGCCAT/ATGGCT | 1 |  | 2 | 1 |  |  |  | 0 | 4 | 0.0 |
| AGCCCT/AGGGCT | 5 | 2 | 2 |  |  |  |  | 0 | 9 | 0.1 |
| AGCCGG/CCGGCT | 3 |  | 1 |  |  |  |  | 0 | 4 | 0.0 |
| AGCCTC/AGGCTG | 1 |  |  |  |  |  |  | 0 | 1 | 0.0 |
| AGCCTG/AGGCTC |  |  |  |  | 1 |  |  | 1 | 2 | 0.0 |
| AGCGGG/CCCGCT | 1 | 1 |  |  |  |  |  | 0 | 2 | 0.0 |
| AGCTCC/AGCTGG | 6 | 1 |  |  |  |  |  | 0 | 7 | 0.1 |
| AGGATC/ATCCTG |  |  | 1 |  | 1 |  |  | 0 | 2 | 0.0 |
| AGGATG/ATCCTC | 5 |  |  | 2 | 1 |  |  | 0 | 8 | 0.1 |
| AGGCAT/ATGCCT | 2 | 1 |  |  |  |  |  | 0 | 3 | 0.0 |
| AGGCCC/CCTGGG | 1 |  |  |  |  |  |  | 0 | 1 | 0.0 |
| AGGCCG/CCTCGG | 4 |  |  |  |  |  |  | 0 | 4 | 0.0 |
| AGGCGG/CCGCCT | 3 |  |  |  |  |  |  | 0 | 3 | 0.0 |
| AGGGAT/ATCCCT | 3 | 1 |  | 1 |  |  |  | 0 | 5 | 0.0 |
| AGGGCC/CCCTGG | 3 |  |  |  |  |  |  | 0 | 3 | 0.0 |
| AGGGCG/CCCTCG | 2 |  | 1 |  |  |  |  | 0 | 3 | 0.0 |
| AGGGGC/CCCCTG | 1 |  | 1 |  |  |  |  | 0 | 2 | 0.0 |
| ATATCC/ATATGG | 5 |  |  | 1 |  |  |  | 0 | 6 | 0.0 |
| ATCATG/ATCATG | 1 |  |  |  |  |  |  | 0 | 1 | 0.0 |
| ATCCCC/ATGGGG | 4 | 1 | 1 |  | 1 |  |  | 0 | 7 | 0.1 |
| ATCCCG/ATCGGG | 3 | 1 |  |  |  |  | 1 | 0 | 5 | 0.0 |
| ATCGCC/ATGGCG | 5 |  |  |  |  |  | 1 | 0 | 6 | 0.0 |
| ATCGGC/ATGCCG | 1 | 1 |  |  |  |  |  | 0 | 2 | 0.0 |
| ATGCCC/ATGGGC | 1 |  |  |  |  |  |  | 0 | 1 | 0.0 |
| CCCCCG/CGGGGG |  | 1 |  |  |  |  |  | 0 | 1 | 0.0 |
| CCCCGG/CCGGGG | 1 |  |  |  |  |  |  | 0 | 1 | 0.0 |
